# Longitudinal HIV sequencing reveals reservoir expression leading to decay which is obscured by clonal expansion

**DOI:** 10.1038/s41467-019-08431-7

**Published:** 2019-02-13

**Authors:** Marilia Rita Pinzone, D. Jake VanBelzen, Sam Weissman, Maria Paola Bertuccio, LaMont Cannon, Emmanuele Venanzi-Rullo, Stephen Migueles, R. Brad Jones, Talia Mota, Sarah B. Joseph, Kevin Groen, Alexander O. Pasternak, Wei-Ting Hwang, Brad Sherman, Anastasios Vourekas, Giuseppe Nunnari, Una O’Doherty

**Affiliations:** 1 Department of Pathology and Laboratory Medicine, University of Pennsylvania, Philadelphia, 19104 PA USA; 2 Department of Molecular Biosciences, Northwestern University, Evanston, 60201 IL USA; 3 Department of Clinical and Experimental Medicine, Unit of Infectious Diseases, University of Messina, Messina, 98124 Italy; 4 Laboratory of Immunoregulation, National Institutes of Allergy & Infectious Diseases, National Institutes of Health, Bethesda, 20892 MD USA; 5 Infectious Disease Division, Weill Cornell Medical College, New York, 10065 NY USA; 6 Department of Microbiology and Immunology, University of North Carolina at Chapel Hill, Chapel Hill, 27599 NC USA; 7 Laboratory of Experimental Virology, Department of Medical Microbiology, Academic Medical Center, University of Amsterdam, Amsterdam, 1105 The Netherlands; 8 Department of Biostatistics, Epidemiology and Informatics, University of Pennsylvania, Philadelphia, 19104 PA USA; 9 Laboratory of Human Retrovirology and Immunoinformatics, Frederick National Laboratories for Cancer Research, Leidos Biomedical Research Inc., supporting the Division of Clinical Research, NIAID, Frederick, 21702 MD USA

## Abstract

After initiating antiretroviral therapy (ART), a rapid decline in HIV viral load is followed by a long period of undetectable viremia. Viral outgrowth assay suggests the reservoir continues to decline slowly. Here, we use full-length sequencing to longitudinally study the proviral landscape of four subjects on ART to investigate the selective pressures influencing the dynamics of the treatment-resistant HIV reservoir. We find intact and defective proviruses that contain genetic elements favoring efficient protein expression decrease over time. Moreover, proviruses that lack these genetic elements, yet contain strong donor splice sequences, increase relatively to other defective proviruses, especially among clones. Our work suggests that HIV expression occurs to a significant extent during ART and results in HIV clearance, but this is obscured by the expansion of proviral clones. Paradoxically, clonal expansion may also be enhanced by HIV expression that leads to splicing between HIV donor splice sites and downstream human exons.

## Abstract (web summary)

How HIV reservoirs are shaped over time on antiviral therapy is poorly understood. Here, the authors analyze the dynamics of the HIV reservoir by longitudinal proviral sequencing revealing that HIV reservoir expression can contribute to its clearance and paradoxically even to its persistence.

## Introduction

The advent of antiretroviral therapy (ART) revealed a treatment-resistant reservoir of HIV proviruses requiring life-long therapy^1^. Pioneering work has shown that the HIV reservoir has a very slow rate of decay. Estimates of reservoir decay suggested a half-life of 44 months using Quantitative Viral Outgrowth Assay (QVOA)^1,2^. However, these measurements were indirect, and their error was sufficiently large that the precise half-life of the reservoir in individual subjects was uncertain. Differentiating error due to assay inconsistency versus biological variation is difficult. If biological variation is prominent, a subset of subjects may have significant reservoir decline while others may not. For this reason, it becomes essential to robustly measure each individual’s reservoir decay rate, especially in cure studies. This biological variation could arise for multiple reasons, including variable ART compliance or biological differences in the host or pathogen.

Viral nucleic acid measurements have been used as a surrogate for HIV reservoir size, as some measures have shown significant correlations with QVOA^3^. Longitudinal studies suggest HIV DNA is relatively stable after the first few years of ART^4^. However, HIV DNA measurements suffer from the presence of defective proviruses, which constitute the majority of the total DNA; thus, while the intra-assay variation for PCR is small, the variable and largely unknown frequency of defective proviruses^5–9^ results in precise but inaccurate estimates of replication-competent reservoir size. As a consequence, large changes in replication-competent proviruses may be masked by defective proviral DNA. Moreover, selective pressures on defective DNA may be different than selective pressures on intact proviruses^10^, and thus HIV DNA measures may not be an appropriate way to longitudinally monitor reservoir dynamics.

Monitoring the frequency of individual proviral sequences over time in the presence of ART could reveal positive and negative selective pressures that act on infected cells. Furthermore, such an approach would differentiate between replication-competent and defective proviruses, allowing for a direct calculation of decay of the replication-competent reservoir. While such an approach is currently not feasible for all HIV-infected individuals due to limited throughput and cost, in-depth study of a subset of subjects might provide new insights into reservoir dynamics as well as the effect of the host on reservoir persistence.

We employed limiting dilution polymerase chain reaction (PCR) followed by DNA sequencing to obtain full-length sequences of integrated HIV proviruses in four subjects on suppressive ART over time. We provide evidence that both intact and defective proviruses that contain genetic elements that favor protein expression are under negative selective pressure. Interestingly, defective proviruses that lack these genetic elements, but encode a strong donor splice sequence, are under relative positive selective pressure. We also show significant biological variation in reservoir decay in two of these individuals. In this case, clonal expansion represents an important factor contributing to slower decay. An important implication from our analysis is that the replication-competent reservoir of intact proviruses is under more negative selection than defective proviruses, suggesting that the majority of the replication-competent reservoir is expressed over time.

## Results

### Longitudinal parameters of four subjects on ART

**Fig. 1:**
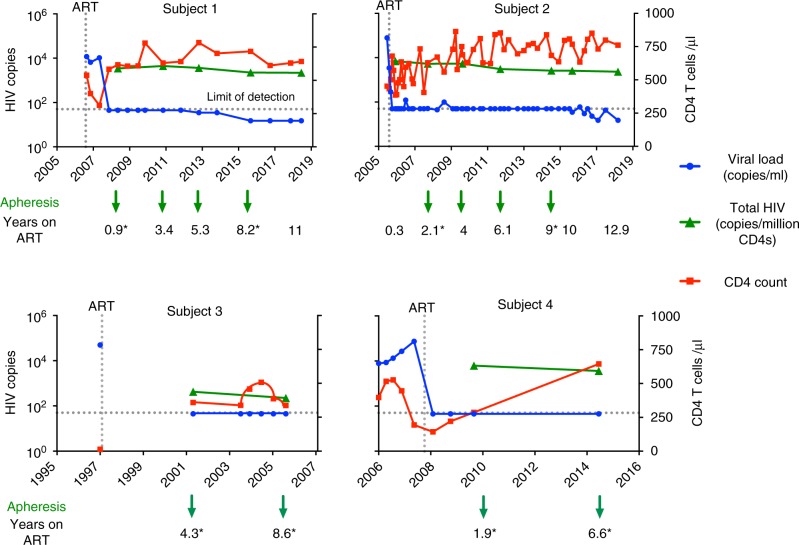
Longitudinal parameters of four subjects over time on ART. Longitudinal levels of plasma HIV-1 RNA (blue), total HIV DNA (green), and CD4 T cell counts (red). For each subject, peripheral blood mononuclear cells (PBMCs) were collected by apheresis at the time points indicated in the graph. The arrows identify the time points we used for sequencing of both intact and defective proviruses, while for the remaining time points only near-full-length proviruses were sequenced. Asteriks identify the time points used for the deletion maps in Fig. 4. Total HIV DNA was quantified by primers binding to the long terminal repeat region of HIV-1. Values are normalized to CD4 T cell count and presented as log copies of HIV per million CD4 T cells. HIV RNA is presented as copies per ml blood

We wanted to assess the decay rate of intact and defective proviruses by combining proviral sequencing with PCR measurements of HIV DNA levels. We identified two subjects with detailed clinical histories (Supplementary Table 1) and sufficient peripheral blood mononuclear cell (PBMC) aliquots spanning more than a decade after achieving virological suppression. For both subjects, total and integrated HIV DNA were assessed at multiple intervals during the first 11–13 years of ART (Fig. 1 and Supplementary Tables 1–3). Viral load and CD4 T cell count were repeatedly assessed in both subjects over the study period. For Subject 1, viral load was always below the detection limit of the diagnostic assay (<50 copies or <20 copies/ml). For Subject 2, the majority of viral load measurements were below the detection limit, with a few episodes of low-level viremia detected after 9 years of continuous virological suppression on ART (Fig. 1). There was a slight decline in total and integrated HIV DNA over the study period, in general less than a two-fold change by any measure (i.e. normalized to CD4, PBMC or per unit volume; Supplementary Tables 2 and 3). In conclusion, RNA and DNA measurements including total and integrated HIV DNA decreased minimally over time, suggesting minimal change in reservoir size. Two additional subjects were also included for whom two time points were available (Fig. 1 and Supplementary Tables 1–3).

### Dynamic changes of intact proviruses imply HIV expression

**Fig. 2:**
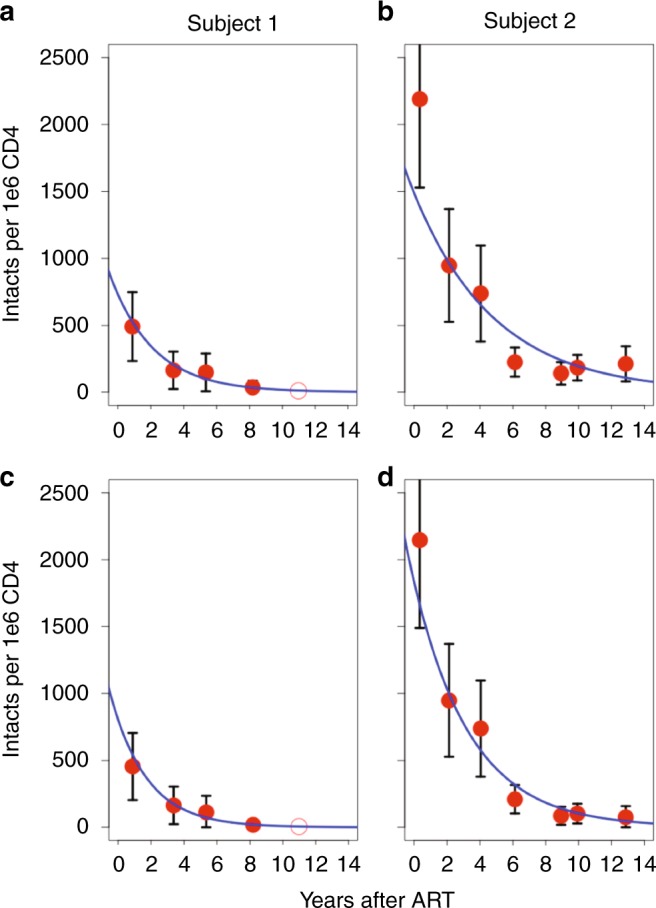
Dynamic changes of intact proviruses over time. **a** Frequency of intact proviruses after initiating treatment for Subject 1 measured by intact copies per million CD4 T cells. Red circles represent intact proviruses calculated by multiplying the concentration of total HIV DNA per CD4 by the frequency of sequenced proviruses that were intact. **b** Frequency of intact proviruses for Subject 2. **c** Frequency of intact proviruses for Subject 1 when counting clones only the first time they were detected in order to minimize the effects of clonal expansion. **d** Frequency of intact proviruses in Subject 2 with clones counted only once, when they first appeared. We included five time points for Subject 1 and 7 for Subject 2. For Subject 1, we did not identify any intact provirus in 2018, and therefore this time point is presented as an open circle. Black bars signify 95 percent confidence interval of the mean based on a binomial process with approximately 100 sequences per time point. The blue line is the estimated decay based on a exponential decay model

To estimate the decay rate of the HIV reservoir, we performed limiting dilution full-length PCR at multiple time points in Subjects 1 and 2 followed by proviral sequencing of the full-length amplicons to determine if they were intact. Our criteria for an intact provirus were the presence of nine open reading frames (ORFs) and 3–4 stem loops at the psi packaging site as well as several critical donor and acceptor splice sequences^11–13^ and the Rev-responsive element (RRE) sequence^14^, as detailed in the Methods section. When we plotted the frequency of intact proviruses over time, we noticed substantial decay (Fig. 2a, b), in contrast to total HIV DNA, which was relatively constant over the same time frame (Fig. 1). For Subject 1, we found the exponential decay rate was −0.38/year with a half-life of 1.8 years. For the purpose of modeling, time 0 was the moment the subject was placed on ART. Using the best-fit exponential decay curve, we predicted that Subject 1 had an estimated 734 intact proviruses per million CD4 T cells contributing to his reservoir at the time he was placed on ART. After 11 years of ART, we estimated that 98% of the cells with intact proviruses were cleared (Fig. 2a). Subject 2 had a slower decay rate (−0.2/year) with a half-life of 3.4 years, and after 11 years the number of intact proviruses declined from an estimated 1490 to 158 per million CD4 T cells (Fig. 2b). This slower decay of intact proviruses in Subject 2 may be due to multiple reasons, including clonal expansion, ongoing replication^15^, and redistribution of infected cells from lymphoid tissue.

**Fig. 3:**
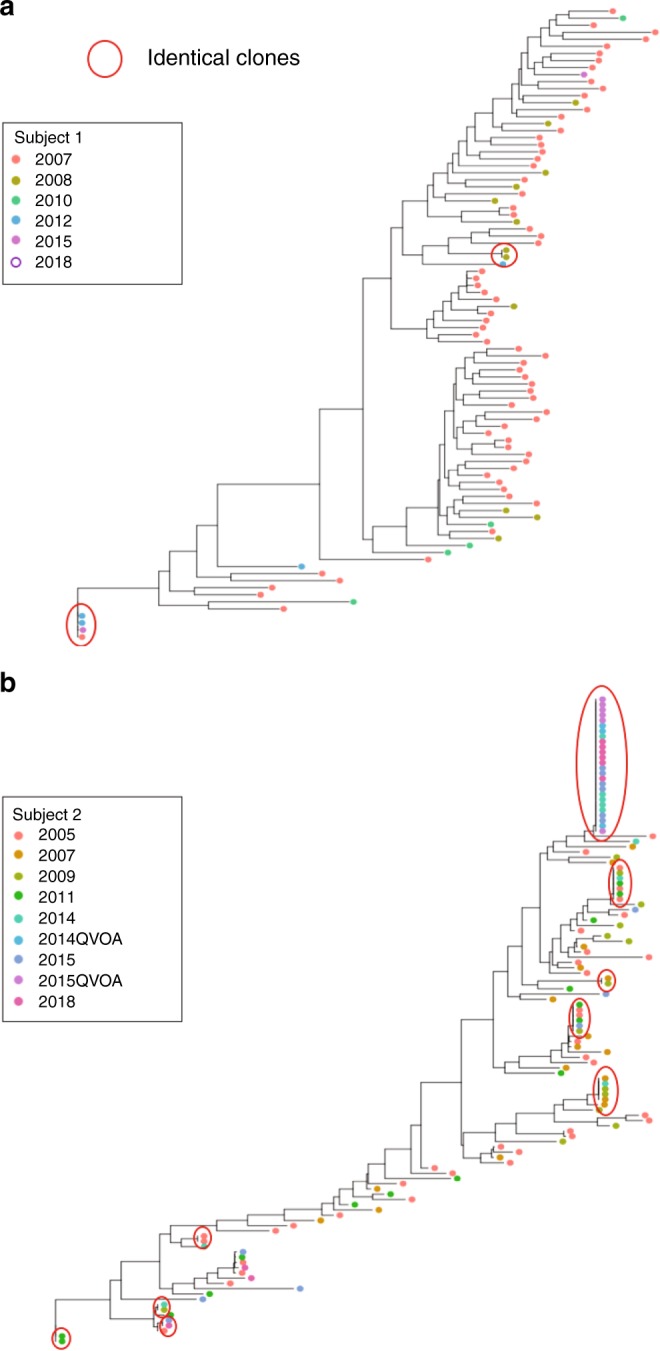
Phylogenetic tree of intact proviruses for Subjects 1 and 2. **a** Phylogenetic tree of intact proviruses for Subject 1 (*n* = 90). Branch lengths are proportional to genetic distance to a consensus sequence for the sequences graphed in the tree. Identical clones are indicated within the red circles. Consensus sequences were generated for each subject and used to root the tree. **b** Phylogenetic tree of intact proviruses for Subject 2 (*n* = 123). Circled clones represent identical intact proviruses. Notably, we included 67 sequences from 2007 for Subject 1, at which time he was in the first phase of viral decay; for this reason, we excluded this time point in our model of intact proviral decay^79^. For Subject 1, no intact proviruses were identified in the sample from 2018, and therefore this time point is presented as an open circle

To investigate the role of clonal expansion in proviral decay over time, we aligned intact proviruses and generated a phylogenetic tree for Subjects 1 and 2 independently (Fig. 3a, b). We noticed that there was no increase in sequence diversity over time, consistent with no replication^16–20^ or minimal replication^21–25^ on ART. We identified several identical sequences in the intact tree, suggesting clonal expansion of intact proviruses^6,9,26–32^. For Subject 1, there were occasional identical intact clones, but they did not appear to increase in frequency over time. There was one pair of identical sequences in 2008, and another distinct pair was identified in 2012 which was also detected once in 2015. While identical sequences only accounted for ~22% of intact proviruses in Subject 1, in 2015 half of the intact proviruses (1 out of 2) had been sampled at earlier time points. We found no intact proviruses in the 2018 sample from Subject 1 (Fig. 3a). For Subject 2, we identified nine distinct clones of intact proviruses which increased in prevalence over time. One identical clone emerged ~9 years after starting ART (2014) in Subject 2 and persisted in samples from 2015 and 2018. This proviral clone was capable of releasing infectious virus as measured by QVOA (Fig. 3b). The presence of identical sequences is suggestive of clonal expansion of cells harboring intact proviruses, a recently supported phenomenon^6,9,26–32^ which may contribute significantly to the maintenance of the intact reservoir. To minimize the effect of clonal expansion on reservoir decay, we next counted each clone only once, at the time it first appeared (Fig. 2c, d), assuming proviruses with identical sequences were clones. We found that this led to a greater fit to the exponential model, suggesting that when the effects of clonal expansion are reduced the resulting dynamics more closely follow an exponential decay. Given the substantial decay of intact proviruses when clonal expansion is minimized, our data suggest that a significant portion of the reservoir is expressed over time, consistent with recent literature^33–35^ as well as intracellular RNA measures in both subjects (Supplementary Table 2).

### Role of splicing on the proviral landscape

**Fig. 4:**
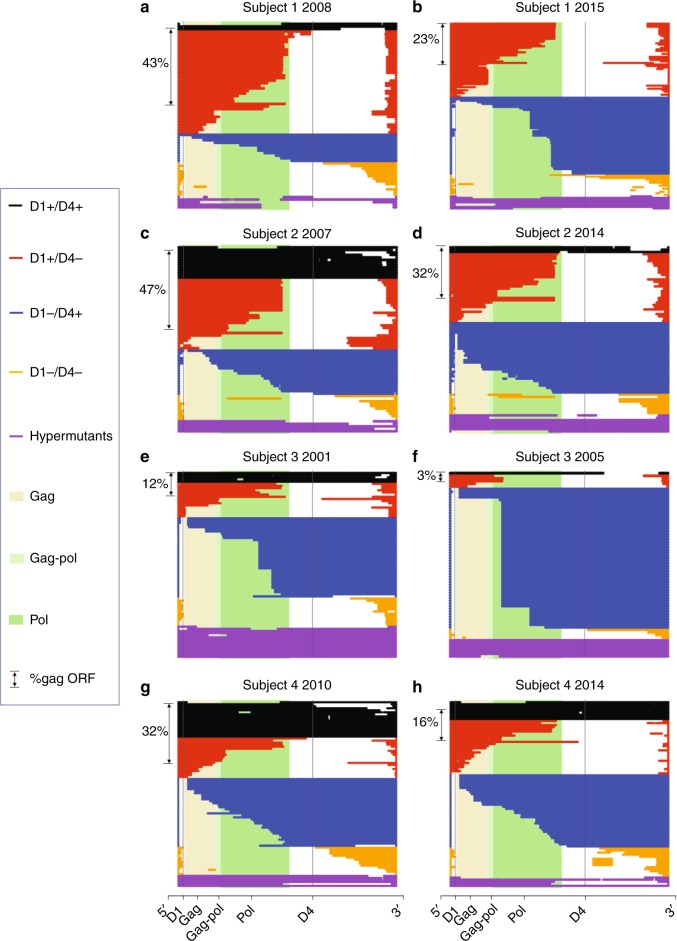
Deletion analysis reveals a role for splicing in reservoir dynamics. **a** Defective proviruses from an apheresis sample collected from Subject 1 in 2008 (~1 year of ART) are aligned to HXB2. In order, from top to bottom, black proviruses are D1+ D4+, red proviruses are D1+ D4−, blue proviruses are D1− D4+, and gold proviruses are D1−D4-. Hypermutated proviruses are represented in purple. The shaded beige, light green, and dark green regions correspond to the gag, gag-pol, and pol regions of HXB2, respectively. On the left side of panels **a**–**h** we show the percentage of defective proviruses containing a complete Gag ORF. **b** Defective proviruses from Subject 1 for the apheresis sample collected in 2015 (~8 years of ART). Proviruses are graphed on the same scale to demonstrate how the proportion of each type of defective proviruses changed from first to last time point. **c**, **d** Defective proviruses from Subject 2 for the apheresis sample collected in 2007 (~2 years of ART) and in 2014 (~9 years of ART). **e**, **f** Defective proviruses from Subject 3 for the apheresis sample collected in 2001 (~4 years of ART) and in 2005 (~9 years of ART). **g**, **h** Defective proviruses from Subject 4 for the apheresis sample collected in 2010 (~2 years of ART) and in 2014 (~7 years of ART). The time points used for the deletion maps are identified by asterisks in Fig. 1. The first black provirus depicted in **a** contains a D1 and terminates within the gag ORF, but this is obscured due to imperfect R-coded filtering

Our results of minimal changes in HIV DNA yet a significant decline in intact proviruses over time led us to investigate the dynamics of defective proviruses in the reservoir. Importantly, defective proviruses are not expected to be subject to selective pressures that arise through ongoing replication. We restricted our study to four similar time points for Subjects 1 and 2, spanning nearly 10 years on ART and 2 time points for Subjects 3 and 4 (identified by arrows in Fig. 1). We performed full-length proviral sequencing of every amplicon obtained at limiting dilution. In this way, we sequenced over 1400 individual proviruses and performed de novo assembly to generate contiguous sequences. Evaluation of the de novo assembled proviruses showed a predominance of large deletions, in agreement with other studies^5–10^. Supplementary Figure 1 shows the distribution of the sequenced proviruses according to the number of ORFs. We observed that proviruses with nine complete ORFs tended to contract over time while proviruses with 0 ORFs did not. A detailed analysis of ORF selection showed that proviruses with an intact HIV Gag ORF were negatively selected, but no other clear pattern emerged (Fig. 4). Initially, this seemed counterintuitive since Gag is less toxic than other HIV proteins. This led us to examine the deletion maps of the subjects which revealed that splicing could play an important role in selection.

To visually inspect for selection pressures that might be exerted on deleted proviruses, we first aligned all defective proviruses from two time points for Subject 1 (Fig. 4a, b) and Subject 2 (Fig. 4c, d) identified by asterisk in Fig. 1 as well as Subjects 3 (Fig. 4e, f) and 4 (Fig. 4g, h) to the reference HXB2. Next, we grouped the deleted proviruses into categories based on the presence or absence of the donor splice sites 1 (D1) and 4 (D4). D1 and D4 are unique among HIV splice sites for their strong ability to interact with U1 small nuclear ribonucleoprotein (snRNP) and splice with a downstream acceptor. The other splice donor and acceptor sites of HIV are all considered weak^12,36^. We categorized deleted proviruses as follows: D1+D4+ (black), D1+D4− (red), D1−D4+ (blue), D1−D4− (gold), and hypermutated proviruses (purple). Upon inspection, we found that the proportion of D1+D4+ (black) proviruses contracted the most. These proviruses had small deletions but retained D1 and D4. In addition, a subset of the D1+D4− (red) proviruses, which contain 3′ deletions that encompass D4, also appeared to contract over time, especially in Subject 1. Interestingly, we found that a large fraction of these proviruses had the genetic potential to express HIV Gag and occasionally HIV Pol (Fig. 4a–d). Notably, despite the fact that the red proviruses lack the RRE, there is evidence that nuclear export of Gag can be Rev-independent^37^. Thus, we hypothesize that Gag transcripts can reach the cytoplasm and be translated even in the absence of Rev. The fraction of D1−D4− (gold) and hypermutated (purple) proviruses did not significantly change over time. Neither of these proviruses would be expected to express functional HIV proteins. Overall, it seems the potential to express HIV proteins exerts a negative selection pressure on the HIV proviral landscape.

One category of proviruses increased relatively over time. These proviruses are represented in blue in Fig. 4. All blue proviruses contained a deletion in the 5′ end and are missing the major splice donor site D1, but they all preserved D4 (D1−D4+). On inspection, it became clear that at the later time points several of the blue proviruses were identical sequences and represented defective proviral clones. These proviruses are not expected to express HIV Gag/Pol efficiently because they lack the canonical AUG for Gag/Pol. For this category of defective proviruses, the deletion begins in front of the four stem loops of the psi packaging site and ends before D4. This is true by definition because D1 is located within the second stem loop of the packaging site. Importantly, D1 is utilized in the canonical splice pathway for all the proteins besides Gag/Pol which are encoded on the 3′ end of HIV. Given that all canonical spliced and unspliced forms of HIV have by design the same 5′ untranslated region (5′UTR) with extensive secondary structure, including the Trans-activation response element (TAR) and the four packaging stem loops^38^, it is reasonable to assume that HIV has evolved to efficiently translate proteins when the entire 5′UTR is placed next to the favored AUG. It follows that truncating the 5′UTR would likely make HIV translation less efficient. Thus, inefficient protein expression may provide one mechanism for the relative increase over time of D1−D4+ proviruses. Importantly, we observed similar patterns in two additional subjects for which only two time points were available for analysis (Fig. 4e–h).

Immune evasion may also contribute to the relative preservation of D1−D4+ proviruses. All of the D1−D4+ proviruses also contain a complete ORF for Nef, which could provide a mechanism of immune evasion^39–43^. Nef has been shown to downregulate MHC and to provide protection from CTL clearance^39–43^. These changes, while still present in Subject 2, tended to be less dramatic as compared to the other subjects. This could be due to weaker immune responses in Subject 2, which in turn would be consistent with his clinical history, characterized by HLA B35 haplotype and a rapid drop in CD4 T cell count [down to 0 CD4 T cells six years after diagnosis (Supplementary Table 1)]. Thus, D1−D4+ proviral clones may be relatively preserved because they evade the immune system by Nef.

### Two opposing forces shape the proviral landscape

**Fig. 5:**
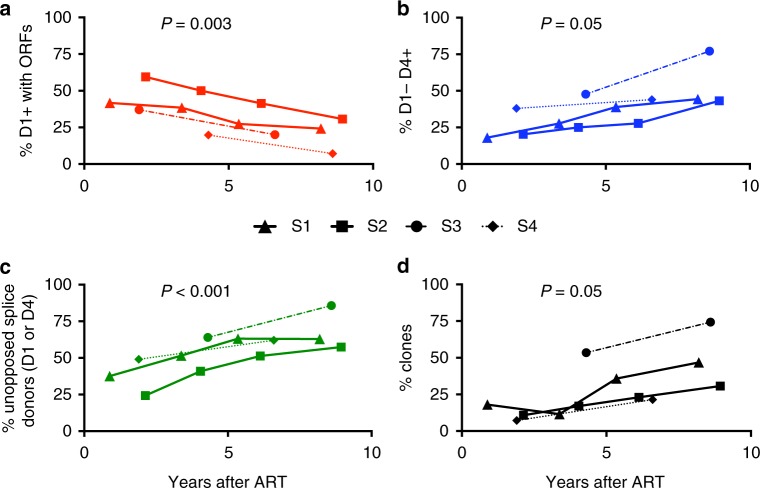
Relative changes in the major splice sites donors reveal selection pressures. **a** Percentage of defective proviruses with D1 splice site and at least one ORF over time on ART (red). **b** Percentage of intact D4 splice site sequence in defective proviruses lacking 5′ D1 at the same time points. These proviruses are predicted not to express proteins efficiently due to a truncated 5′UTR (blue). **c** Percentage of defective proviruses with unopposed strong donor splice site, i.e. D1+ without ORFs or D1−D4+ at the same time points (green). **d** Percentage of clones over time in defective proviruses (black). Estimations of a common slope for these data were done using a linear random-effects regression model, assuming each subject had a different intercept at the initiation of ART. To test for the statistical significance of this effect, a statistical analysis based on a type III Anova was performed. The time points used for the analysis of the deleted proviruses are identified by arrows in Fig. 1

To probe if expression potential was important for selective pressure we investigated whether defective proviruses with an intact D1 sequence and at least one intact ORF were selected against and thus declined over time. We required the presence of D1 and at least one intact ORF because proteins that are expressed on the 3′end utilize this sequence to make the canonical spliced products that encode all proteins except Gag/Pol^12,13^. The majority of proviruses meeting these criteria contained an intact Gag ORF (Fig. 4). We excluded intact proviruses which clearly decreased over time from this analysis to concentrate on the pressures exerted on defective proviruses. By using a linear random-effects regression model, we found a relative decline in defective proviruses with a D1 splice site and at least one intact ORF in the four subjects (*P *= 0.003 by type III Anova; Fig. 5a). Thus, our data suggest that the potential to express proteins even among defective proviruses correlates with relative clearance of proviral DNA.

We next asked if the presence of a strong D4 in the absence of D1 could explain the relative increase of defective proviruses containing complete ORFs in the 3′end over time. We reasoned that the absence of D1 in a provirus with a large 5′ deletion would truncate the 5′UTR and thereby hinder expression of any of the HIV proteins translated from spliced RNAs^12,13^. Moreover, the vast majority of D1−D4+ proviruses contain an intact ORF for Nef such that if proteins were made, we would expect Nef to be prominent among them. Given that Nef provides a mechanism for immune evasion^39–43^ this might contribute to positive selection. To test this hypothesis, we plotted the presence of D4 without D1 over time among defective proviruses in the four subjects and found a significant increase (*P* = 0.05 by type III Anova), consistent with relative positive selection for proviruses with strong donor splice sites without strong potential for HIV protein expression (Fig. 5b).

We then asked if the presence of a strong donor splice site (D1 or D4) might drive the relative increase in deleted proviruses when genetic elements that favor protein expression are missing, potentially by enhancing clonal expansion. We defined those proviruses that lack genetic elements to promote protein expression (D1−D4+ and D1+ without ORFs) as proviruses with an unopposed strong donor splice site. In other words, the term unopposed indicates proviruses without genetic elements that favor protein expression. We found a correlation between the percentage of unopposed splice sites and time (*P* < 0.001 by type III Anova; Fig. 5c). In summary, the number of defective proviruses that contained unopposed strong donor splice sites increased over time which likely reflects proliferation of cells containing these defective proviruses.

We next plotted the frequency of all clones over time as a proportion of the reservoir. In all subjects we observed the expansion of proviral clones over time (*P* = 0.05 by type III Anova; Fig. 5d). Clones steadily increased relative to other defective proviruses over time, consistent with several recent studies^29–31,44,45^. Splicing may be one driver of clonal expansion since a higher percentage of clones contained unopposed splice sites than defective proviruses in general (Supplementary Table 4, *P* = 0.02 by Wilcoxon signed-rank test). Notably, the absolute number of defective proviruses with unopposed splicing potential as well as clones did not change over time on ART (Supplementary Fig. 2). This suggests that the majority of clonal expansion occurs before or near the time of ART initiation (Supplementary Fig. 2). Nonetheless, our data are consistent with other studies^32^ showing that individual proviral clones wax and wane during ART, indicating that clonal expansion continues even after ART initiation, possibly at a slower rate (Supplementary Fig. 3).

### HIV expression can lead to aberrant splicing

**Fig. 6:**
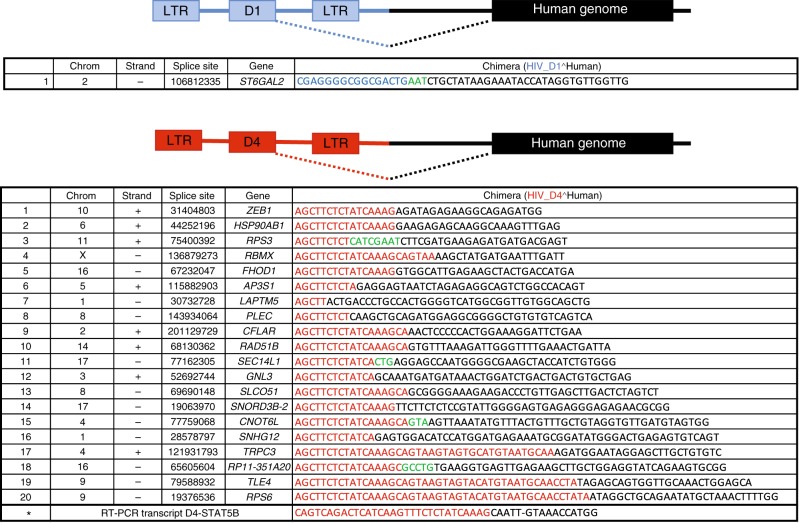
HIV expression leads to chimeric transcripts between D1 or D4 and human genes. RNA-seq was performed using in vitro-infected resting cells harvested at day 7 after infection. The sequences of the chimeric transcripts between HIV D1 (blue) and human genes (black) are shown in the top panel, while those between HIV D4 (red) and human genes (black) are shown in the bottom panel. We marked in green the sequences we could not map to either HIV or the human genome.The sequence marked with an asterisk was retrieved by RT-PCR and Sanger sequencing of in vitro-infected CD4 T cells

In order to investigate the biological evidence supporting our findings, we measured the frequency of cells that contained unspliced HIV RNA and multispliced (ms) HIV RNA in Subjects 1 and 2 (Supplementary Table 2). We found between 10% and 30% of proviruses were actively transcribing at the four time points tested consistent with^33,34^. A much smaller fraction of cells had detectable tat/rev ms HIV RNA. Thus, the potential to splice to downstream oncogenes exists. We then measured the frequency of splicing between D1 or D4 and downstream human genes in our in vitro model of resting T cell infection. In a recent study^46^, D1 was shown to splice to the human oncogene STAT5B, suggesting a potential mechanism that HIV can exploit for its own expansion by integrating into genes that promote cell division. This finding as well as our own results led us to investigate whether D1 or D4 could splice to downstream exons in infected CD4 T cells. We first performed RT-PCR on 600 ng RNA recovered from in vitro-infected CD4 T cells. After two rounds of PCR, we were able to detect and confirm by Sanger sequencing the presence of D4-STAT5B chimeric transcripts (Fig. 6), but not D1-STAT5B transcripts. We then performed RNA-seq on four million in vitro-infected CD4 T cells that were cultured for 7 days in the presence of IL-7 and found that splicing occurred more frequently between D4 and downstream exons in comparison to D1 (20 to 1 ratio; Fig. 6). While our experiment does not address selection, our data are consistent with the idea that splicing between D4 and downstream exons could provide a mechanism to induce cell division. In turn, this may explain why D1−D4+ proviruses are enriched relative to other proviruses over time.

## Discussion

Our longitudinal study of proviral sequences reveals that two opposing forces simultaneously exert negative and relative positive selection pressures on cells containing proviral DNA in HIV-infected individuals on ART. Specifically, we found that proviruses with both an intact 5′UTR and ORFs declined over time while proviruses with strong donor splice sites and a truncated 5′UTR increased relatively over time. Consistent with recent studies^6,9,26–34^, we also found evidence of clonal expansion of intact proviruses. We speculate that positive selection of these proviruses can be driven by the unique positioning of strong HIV donor splice sites within an intron of an oncogene. Our work has several implications for HIV eradication: first, it provides a mechanism for how intact proviruses can decline over time while proviral DNA levels remain unchanged; second, it gives new insights on forces that might drive clonal expansion; third, it provocatively suggests that the HIV reservoir is likely less resistant to reactivation than generally thought. This has important implications for HIV cure as it suggests that the major hurdle to HIV eradication may not be the invisibility of the reservoir. On the contrary, our results suggest that HIV expression may provide a targetable mechanism for HIV persistence.

We find reservoir expression leads to proviral clearance. Our data show that intact proviruses contracted more rapidly than defective proviruses. This finding suggests that intact proviruses experience stronger negative selection. The strong negative pressure against intact proviruses suggests in turn that the majority of the replication-competent reservoir is expressed over time, despite the small fraction that is detectably expressed at any one moment^33–35,47–50^. Negative selective pressures could be due to immune or viral cytotoxicity. It is generally thought that immune pressure during ART is minimal due to the dramatic drop in the total antigen load^51–53^. However, our study suggests that immune pressure can play a role in shaping the reservoir even during suppressive ART in humans. In rhesus macaques there is evidence that CD8 T cell depletion after ART suppression results in rebound viremia, which is consistent with our interpretation^54^.

Deleted proviruses with genetic elements that promote protein expression contract over time. In addition to intact proviruses, we observed the contraction over time of a subset of defective proviruses. We noticed this subset contained both a full-length 5′UTR and at least one functional ORF and fell into the category of D1+ D4+ or D1+ D4−. The 5′UTR is generally defined as the region between the transcription start site and the canonical AUG start codon. This region contains several regulatory elements that play a role in translation initiation^38^. Subject 2 showed a weaker contraction of proviruses with a full-length 5′UTR and gag ORF. This suggests that Subject 2 exerts less immune pressure than the other subjects, as supported by clinical history of more rapid progression (Supplementary Table 1). The variable contraction of proviruses on ART could be due to variable immune pressure. Consistent with this idea, proviral DNA significantly declines on ART in elite controllers and acutely infected individuals, who have a more robust immune response to HIV^55–61^. On the other hand, proviral DNA decline is variable and difficult to detect in chronically infected individuals who have a weaker immune system^59–61^.

Proviruses with limited expression potential are relatively preserved over time. It seems reasonable to assume that cells infected with proviruses that lack ORFs will experience no immune pressure and those that have reduced genetic potential for protein expression would be relatively preserved because they would be less visible to the immune system. Specifically, D1−D4+ proviruses should also be relatively protected from immune clearance as we expect them to be expressed at lower levels since they have a truncated 5′UTR. Interestingly, the D1−D4+ proviruses have deletions that start in front of the HIV packaging site stem loops (downstream of TAR) and end somewhere in gag/pol. While a role for TAR in translation has been supported^38^, further work on the stem loops is needed to demonstrate if they have a role in translational control of HIV^62^. It should also be mentioned that immune evasion provides an additional (not mutually exclusive) mechanism for D1−D4+ preservation. The vast majority of D1−D4+ proviruses contain HIV Nef, which may contribute to immune evasion^39–43,63^.

Proviruses that lack elements to promote protein expression but retain splicing may enhance clonal expansion. Our data in combination with recent work^45,46,64^ suggest that integration of proviruses into introns can stimulate cell division. Long terminal repeat (LTR) transcription can lead to splicing between a strong HIV donor site and an oncogene acceptor site. This in turn could result in higher expression of the oncogene as recently described^46^. Our work advances the findings of Cesana et al.^46^ by showing that unopposed strong donor splice sequences correlate with relative proviral clonal expansion in vivo and by demonstrating the contribution of D4, not just D1 (Figs. 4–6, Supplementary Fig. 2, Supplementary Table 4). Our hypothesis is further supported by the presence of chimeric transcripts between HIV and human genes (Fig. 6). In the context of recent literature, splicing in the absence of immune clearance provides a mechanism for how HIV integration near an oncogene can lead to clonal expansion^45,46,65,64^.

Contraction and expansion forces could be applied to all proviral clones including intact ones. Notably, if HIV inserts within an intron of an oncogene it seems likely that with robust LTR transcription there could a mixture of canonical HIV RNA forms leading to HIV protein expression and aberrant splicing to downstream exons. Cell division would occur if a strong D1 or D4 splice sequence spliced to an exon of an oncogene, leading to forced expression of the oncogene. On the other hand, negative selection pressure may occur if the provirus expresses HIV proteins that could lead to immune-mediated or viral cytotoxicity. In other words, a D1+ D4− provirus could express proteins and also provide a D1 to splice to an oncogene. In fact, this could apply to all categories of proviruses that have the potential to express HIV proteins as well as splice, including the intact ones. Notably, intact proviral clones appear to emerge after many years of ART when the immune system wanes. It seems logical that intact proviral clones would proliferate without being cleared by the waning immune system. If true, this would suggest that immune clearance plays a greater role than viral cytotoxicity in the elimination of proviral clones.

The timing and drivers of clonal expansion remain unclear. One limitation of our approach is that we cannot easily quantify the extent of negative and positive selection individually because they are opposing and simultaneous forces. It is possible that clones form predominantly before starting ART or around the time of ART initiation, then emerge after clearance of the proviruses capable of expressing proteins. This is consistent with previous work showing turnover of T cells is many fold higher before ART is initiated^66^. It is also consistent with our own work that shows a steady increase in proviral DNA over time prior to ART^59^ which then plateaus after ART initiation^4,59^. On the other hand, our data show that individual proviral clones wax and wane over time (Supplementary Figs. 2 and 3), suggesting that clonal expansion likely occurs even during ART, perhaps at a lower rate, perhaps driven by sporadic stimuli. Regardless, the enrichment of clones with identical sequences indicates that some proviral expansion occurs through cell division and likely includes episodic expansions and contractions. Our contribution is to provide evidence that a substantial driving force may be due to unique positioning of splicing sites, but several other drivers beyond splicing likely play a role^67^.

Clonal expansion is a newly identified force driving HIV persistence. In our study, we observed that 78% of intact proviruses in Subject 2 were identical at the last time point. This suggests that cell division contributed to the increase in intact proviruses in Subject 2. Previous investigations have shown that HIV preferentially integrates into actively transcribing genes^68^ and can potentially clonally expand without reactivating and triggering immune clearance^69^. Evidence supporting a role for clonal expansion in proviral persistence has been mounting^6,9,26–32,44–46,64,67,70,71^. In one study, the identical intact sequences were more prominent in effector memory (EM) cells^6^ consistent with work showing the persistence over many years of a mutant HIV clone in EM cells^72^. Another study showed that clonally expanded proviruses are important for viral rebound^33^. The accumulation of intact clonal sequences (Fig. 3) in Subject 2 adds to the evidence that clonal expansion plays a role in reservoir persistence and suggests that its contribution is substantial.

An important limitation of our study is the small number of subjects studied as well as the fact that our analysis is comprised of only the circulating white blood cells. Thus, our data do not reflect the selection of proviruses in tissue-resident lymphocytes that do not recirculate. However, as recent studies have shown that the phylogeny of proviruses isolated from lymph nodes and blood is similar, our analysis likely reflects selection pressures exerted on the circulating pool of lymphocytes that travel between the blood, spleen, and lymph nodes^33,34^. Moreover, we cannot rule out that some rounds of ongoing replication (either due to poor ART penetration into some compartments or drug resistance) may have occurred, especially in Subject 2. However, the lack of phylogenetic evidence of ongoing replication and the demonstration that the predominant intact sequences are predicted to be susceptible to ART, combined with the prevalence of clonal expansion, reinforce the notion that cellular expansion, not viral replication, is likely the primary driver of positive selection in individuals on ART.

Taken together, our data suggest that intact HIV proviruses are under stronger negative selective pressure for clearance than defective proviruses. This suggests indirectly that the majority of the reservoir is expressed over several years, implying that lack of HIV expression may not be the main hurdle to reservoir clearance. In fact, the very expression of HIV RNA may play an important role in HIV persistence as RNA transcription is essential for HIV splicing. Splicing in turn may enhance expression of downstream genes involved in cell division and may represent a new target for HIV cure.

## Methods

### Apheresis

Subjects underwent apheresis at the University of Pennsylvania according to protocols #704904, approved by the Institutional Review Board (IRB). Each subject signed an informed written consent to be enrolled in the study. The early time point samples from Subjects 1 and 2 as well as the samples from Subjects 3 and 4 were provided by Dr. Stephen Migueles (National Institute of Health) who follows his institutional protocol with IRB approval.

### DNA isolation and quantification of HIV DNA

DNA was isolated from PBMCs using the Gentra Puregene Cell Kit (Qiagen). HIV DNA was quantified by total HIV against the LTR (primers LTR F and LTR R; Table 1) or gag regions (primers gag F and gag R; Table 1). Integrated HIV DNA was measured using primers Alu F and gag R; Table 1). First-step PCR reactions were cycled using the Nexus Master Cycler (Eppendorf) and qPCR reactions were cycled on a 7500 FAST real-time instrument (ThermoFisher). For total HIV, PCR conditions for the first round were: 95 °C for 2 min; then 95 °C for 15 s, 64 °C for 45 s, 72 °C for 1 min for 12 cycles; and then 72 °C for 10 min. For Alu-gag measurements the following PCR conditions for the first round were used: 95 °C for 2 min; then 95 °C for 15 s, 56 °C for 45 s, 72 °C for 3:30 min for 40 cycles; and then 72 °C for 10 min. Fifteen microliters of the first-round PCR reactions were run on the qPCR instrument using the primers LTR F and LTR R for total HIV LTR and Alu-gag reactions and primers gag F and gag R for total HIV gag measurements (Table 1). PCR conditions were: 95 °C for 15 s; then 95 °C for 10 s, 60 °C for 20 s for 40 cycles.

**Table 1:** List of primers used in the study

*Total HIV LTR*	
LTR F	TTAAGCCTCAATAAAGCTTGCC
LTR R	GTTCGGGCGCCACTGCTAGA
probe (for qPCR)	CCAGAGTCACACAACAGACGGGCACA
*Total HIV gag*	
gag F	AGTTGGAGGACATCAAGCAGCCATGCAAAT
gag R	TGCTATGTCAGTTCCCCTTGGTTCTCT
probe (for qPCR)	ACCATCAATGAGGAAGCTGCAGAATGGG
*Alu-gag assay*	
Alu F	GCCTCCCAAAGTGCTGGGATTACAG
*Cell-associated HIV RNA*	
*Subject 1 preamplification*	
gag1	TCAGCCCAGAAGTAATACCCATGT
gag R	TGCTATGTCAGTTCCCCTTGGTTCTCT
HIV-rev	TCTCGACGCAGGACTCG
rev R	GCTGTCTCCGCTTCTTCCT
*Subject 2 preamplification*	
gag1	TCAGCCCAGAAGTAATACCCATGT
gag R	TGCTATGTCAGTTCCCCTTGGTTCTCT
MS total	GAAGAAGCGGAGACAGCGACGA
MF83	GGATCTGTCTCTGTCTCTCTCTCCACC
*US RNA qPCR (Subjects 1 and 2)*	
gag1	TCAGCCCAGAAGTAATACCCATGT
gag2	CACTGTGTTTAGCATGGTGTTT
gag3 probe	ATTATCAGAAGGAGCCACCCCACAAGA
*Subject 1 MS RNA qPCR (exons 1–4)*	
rev F	AGGACTCGGCTTGCTGAA
rev R	GCTGTCTCCGCTTCTTCCT
rev probe	CACRGCAAGAGGCGAGGGG
*Subject 2 MS RNA qPCR (exons 4–7)*	
Mf84	ACAGTCAGACTCATCAAGTTTCTCTATCAAAGCA
Mf83	GGATCTGTCTCTGTCTCTCTCTCCACC
Ks2-tq probe	TTCCTTCGGGCCTGTCGGGTCCC
*Illumina sequencing primers*	
First PCR F	CCTCAATAAAGCTTGCCTTGAGTGC
First PCR R	CCTAGTTAGCCAGAGAGCTCCCAG
Second PCR F	AAGTAGTGTGTGCCCGTCTGTTGTGTGAC
Second PCR R	GGAAAGTCCCCAGCGGAAAGTCCCTTGTAG
*RT-PCR*	
STAT5B 1	CATTGTTGGCTTCTCGGACC
LTR 2	GAGCTGTCTGGCTAACTAGG
STAT5B 2	GGGCAGCGGTCATACGTG
LTR 3	AGCTTGCCTTGAGTGCTTCA
STAT5B 3	GCTTGGCTTTCAATCCACTG
D4 F	TATGGCAGGAAGAAGCGGAG
*Pacbio sequencing primers*	
PB5HChaviF	CCTTGAGTGCTTCAAGTAGTGTGTGCCCGTCTGT
PB5HChaviR	CTTGCCACACAATCATCACCTGCCAT
VIF1C	GGGTTTATTACAGGGACAGCAGAG
Ofm19	GCACTCAAGGCAAGCTTTATTGAGGCTTA

### Cell-associated HIV RNA measurements

Frequencies of cell-associated HIV RNA+ cells per million PBMC and HIV RNA copy numbers per cell were measured by limiting dilution-duplex seminested qPCR assay that measures unspliced (us) and multispliced (ms) RNA simultaneously in the same RNA aliquot^73^. US RNA for both subjects was measured by a gag assay. MS RNA for Subject 1 was measured by an assay that amplifies exon 1–4 junction, while for Subject 2 it was measured by an assay that amplifies exon 4–7 junction. Different primers for MS RNA were chosen because of primer mismatches. The primers used for these measurements are reported in Table 1.

### Provirus amplification and sequencing

A two-step nested PCR approach was used to reduce non-specific amplification from genomic targets. Primer sets used in both reactions were located within the LTRs and were staggered appropriately to avoid localized LTR amplification as well as LTR-related PCR artifacts while simultaneously capturing nearly the full-length of HIV proviruses (Table 1). We used a long-range and high-fidelity polymerase enzyme for both reactions (Platinum SuperFi PCR Master Mix; ThermoFisher). In the first PCR reaction, PBMC DNA was diluted so that PCR amplification resulted in ≤30% of wells being positive for HIV DNA. The following PCR cycling conditions were used for both rounds: 95 °C for 2 min; then 95 °C for 15 s, 68 °C for 8 min (15 cycles for the first PCR, 40 cycles for the second one); then 72 °C for 5 min. Nested PCR reactions were visualized by gel electrophoresis, and the fraction of reactions containing ≥2 bands were excluded from our analysis as these were often found to contain multiple proviruses. PCR amplicons were purified using the DNA Clean & Concentrator kit (Zymogen) and DNA concentration was measured using the Quant-iT dsDNA Broad Range Assay Kit (ThermoFisher). Amplicons were prepared using the Nextera library preparation kit (Illumina) and sequenced on a MiniSeq System using a Mid-output flow cell (Illumina).

### Sequence assembly and removal of double proviruses

Paired reads were trimmed in the program Geneious using the BBDuk plugin, discarding reads from the adaptor, and then merged using Geneious. Again, the reads were trimmed of those with a quality rating under 30, and those under 115 base pairs in length. The reads were then mapped using BBMap to the HXB2 reference sequence, and the reads that aligned to the HIV sequence were extracted. Provirus contigs were made through de novo assembly of the extracted reads. Contigs generated by Spades, Tadpole, and Trinity de novo assemblers were compared. Accuracy of each assembler was evaluated by: (1) its ability to produce a contig matching the length of the region supported by reads when mapped to an HIV reference (2) reads supporting the generated contig. We selected Spades as our default de novo assembler based on these criteria. Reads were de novo assembled using Spades with default settings. When reads gave rise to multiple non-overlapping contigs, the contigs were concatenated into one sequence. The final contig was then mapped back to HXB2, and annotated with motifs, including splice donor and acceptor sites, and ORFs as described in the Supplementary Methods 2. Finally, in order to determine whether two proviruses had been sequenced together (double proviruses), the extracted reads were aligned to the assembled contig. Double proviruses were identified according to the criteria listed in the Supplementary Methods 1 and discarded from analysis.

### Nomenclature

Intact proviruses were defined as those determined to code for nearly complete psi packaging sites with at least three stem loops (SL2 has to be intact because it contains D1^13^) and nine complete ORFs for all HIV genes. We allowed for truncated Nef and Tat genes as commonly identified in infectious strains of HIV^74^. Nef was allowed to be truncated up to the extent seen in NL4-3. We required the presence of Major Donor Site 1 or a GT dinucleotide cryptic donor site located four nucleotides downstream^13^ (only found in four proviruses) and presence of Major Donor Site 4. We also required the presence of splice Acceptor Site A5, A7, either A4a or A4b or A4c as well as an intact RRE sequence (Supplementary Table 5)^13^. We also accepted the sequence GGTAAGT as well as the canonical donor 1 splice sequence GGTGAGT for the D1 sequence as these sequences binds U1 snRNP equally well^75^. Notably this D1 variant sequence was found in a proviral sequence with no functional ORFs that was present at increasing frequency over time, consistent with clonal expansion.

### Intact provirus decay analysis

Based on intact criteria, the number of intact proviruses per million CD4 T cells was calculated for each subject at multiple time points. To estimate decay parameters, a statistical analysis was performed using a random-effects regression model assuming first-order decay kinetics. Setting time *t* = 0 to be the date when ART was initiated, we were able to estimate the number of intact proviruses at the beginning of treatment, decay rate, as well as their half-life.

### Deletion analysis

Provirus consensus sequences were aligned by MAFFT^76^ using the iterative E-INS-i method with a gap penalty opening penalty of 1.8. This facilitates proper alignment of proviruses of different lengths, which is common among proviruses with deletions. Aligned sequences were then exported to an R software environment using the Seqinr Biological Sequence Retrieval and Analysis package. Once in the R software environment, a program removed base pairs within each subject′s proviruses which were insertions relative to the HXB2 HIV sequence. This allowed the alignment of all proviruses to be standardized in length with base pair indices to HXB2. Then, deletions with length more than 100 base pairs were recorded, and a graph was made in R which showed each sequence plotted against the base pair numbers of HXB2, with deletions shown (Fig. 4).

### Identification of hypermutant sequences

To identify hypermutant HIV sequences, all proviruses for each individual were aligned using MAFFT with the E-INSi algorithm and a 1.8 gap penalty, and an intact HIV sequence was selected as the reference. The aligned proviruses were checked against the reference for hypermutants using the LANL Hypermut 2 program. The provirus with the lowest chance of being a hypermutant as determined by the Hypermut program was selected as the reference, and once again Hypermut 2 was run on the alignment. Proviruses determined to be hypermutant with *P* < 0.05 were counted as hypermutant ones.

### Phylogenies and identification of potential clones

Intact proviral sequences were aligned using MAFFT^76^ with the G-INSi algorithm with a 1.8 gap penalty. A maximum likelihood tree was constructed using PHYML with the general time reversible substitution model, using both SPR and NNI optimization methods for topology, four substitution rate categories, and an estimated transition/transversion ratio, proportion of invariable sites, and gamma distribution parameter^77^.

As described above, all intact proviruses were aligned in MAFFT using the E-INSi algorithm to find potential clones, defined as proviruses with the same sequence and similar length. We first trimmed the entire 5′ LTR and the 3′ LTR up to the end of nef. This was done to remove any ambiguous nucleotides due to poor assembly at both ends of the proviruses. We then created a phylogeny of the intact proviruses as described above, except those with inversions and large insertions. Proviruses that clustered closely in the phylogeny were then individually aligned with each other and manually checked for identical sequences (with sequence differences highlighted by Geneious). Clones were checked for a second time, this time once again aligning all proviruses with MAFFT and then among proviruses of a similar length manually identifying proviruses with identical sequences.

### QVOA assay

For Subject 2, we set up a QVOA using PBMCs from 2014 and 2015. Total CD4 T cells were negatively selected from PBMCs using the EasySepTM Human CD4+ T cell Isolation Kit (Stemcell technologies) and cultured at limiting dilutions in RPMI supplemented with 50 U/ml IL-2, IL-15 superagonist (ALT-803; 72 ng/ml; ALTOR), 10% fetal bovine serum, penicillin–streptomycin, l-glutamine, and PHA (2 µg/ml). Allogeneic feeder PBMCs from a healthy donor were irradiated and added to culture. MOLT-4 cells (CRL-1582, ATCC) were added 24 h later and the cells were cultured for 2 weeks with half media changes every 3–4 days. After 2 weeks, supernatants were screened for p24 using NCI Fredrick p24 ELISA kits. IUPMs were calculated using extreme limiting dilution analysis software (Walter and Eliza Hall).

### Sequencing of outgrowth virus populations

Viral RNA was isolated from each p24-positive QVOA well and converted to cDNA using an oligo(dT) primer and Superscript III Reverse Transcriptase (Table 1). For each p24-positive QVOA well, a nearly full-length viral genome (~8845 bases in length) was amplified in two segments using barcoded primers that labeled each amplicon with one of 64 different barcodes. Amplicons were then separately gel purified using the Qiagen MinElute Gel Extraction Kit and SMARTbell Adaptors were added to the amplicons using the template Prep Kit (PacBio). Sixty-four amplicons (each with a different barcode) were pooled into a library and libraries were submitted for PacBio sequencing (movie time of 10 h). The resulting sequences were first grouped by barcode, thus allowing identification of the QVOA well from which a virus was derived and correction of sequencing errors. High-quality sequences were then analyzed using the PacBio Long Amplicon Analysis (LAA) package. The 5′ and 3′ amplicons for the same virus were joined and visually screened to confirm that ORFs were intact.

### RT-PCR and RNA-seq

Resting CD4 T cells of an uninfected donor were isolated and infected with NL4-3 by spinoculation by centrifuging viral supernatant on cells at 1200*g* for 2 h at 25 °C^78^. Cells were cultured for 7 days in the presence of IL-7 20 ng/ml and SQV 1 µM. At day 7, cells were harvested and total RNA was isolated using Trizol. RT-PCR was performed with 600 ng RNA per reaction, with primers binding to the HIV LTR (primer LTR 2) and STAT5B exon 6 (primer STAT5B 1) (Table 1)^46^. The enzymes Superscript III and Platinum Taq polymerase were used for reverse transcription and amplification, respectively. The amplification product was diluted 1:50 and reamplified in two separate reactions with Taq polymerase using nested primers. In one reaction, the nested primers from Cesana et al.^46^ were used to further amplify D1-STAT5B transcripts (primer LTR 3 and STAT5B 2), and in another reaction nested primers were used to amplify D4-STAT5B transcripts (primer D4 F and STAT5B 3; Table 1). To isolate D4-STAT5B, we designed our own D4 and STAT5B primers (Table 1). Our nested STAT5B primer was in exon 2 of STAT5B, closer to the junction between HIV and STAT5B observed by Cesana et al.^46^. Gel electrophoresis was performed on 30% of the amplification product from each reaction, and a band at the expected length was seen for the D4-STAT5B amplicon (210 nt), but not for D1-STAT5B. Repeat PCR was performed on the remaining D4-STAT5B sample to obtain enough sample for Sanger sequencing using the same primers. Thirty percent of the product of this reaction was run on a gel, and the band was excised, purified, and Sanger-sequenced with both the D4 F and STAT5B 3 primers. The transcript sequence mapped to HIV up to the D4 splice site, and then mapped to the STAT5B gene starting at the same junction at the start of exon 2 as seen by Cesana et al.^46^.

For RNA-seq, we used 1 µg of RNA isolated by Trizol from the same in vitro-infected cells. Ribosomal RNA was removed using the Ribo-Zero Gold rRNA Removal Kit (Illumina). The enriched messenger RNA was sequenced on a NextSeq 500/550 instrument after library preparation using the TruSeq Stranded Total RNA kit (Illumina).

### Statistical analysis and graphing

Statistical processes were performed using R^®^, Pass, and Microsoft Excel^®^ softwares. Graphpad Prism^®^ software was used for graphing.

### Code availability

The custom computer codes used for this study will be made available to the interested readers upon request to the corresponding author.

### Reporting Summary

Further information on experimental design is available in the Nature Research Reporting Summary linked to this Article.

## Supplementary Information

Supplementary Information

Peer Review File

Reporting Summary

## Data Availability

All relevant data used in this manuscript are available upon request to the corresponding author. The proviral sequences obtained in the study have been submitted to the NCBI Sequence Read Archive under GenBank accession code MK383384-MK385589.
