# Supplementary material for: Longitudinal HIV sequencing reveals reservoir expression leading to decay which is obscured by clonal expansion
Source: Nat Commun. 2019 Feb 13;10:728. doi: 10.1038/s41467-019-08431-7 (PMC6374386; doi:10.1038/s41467-019-08431-7)
Supplement: Supplementary file 1 — Supplementary Information [file 41467_2019_8431_MOESM1_ESM.pdf]

Longitudinal HIV sequencing reveals reservoir expression leading to decay which is obscured by clonal expansion

Pinzone *et al.*

Supplementary Figures

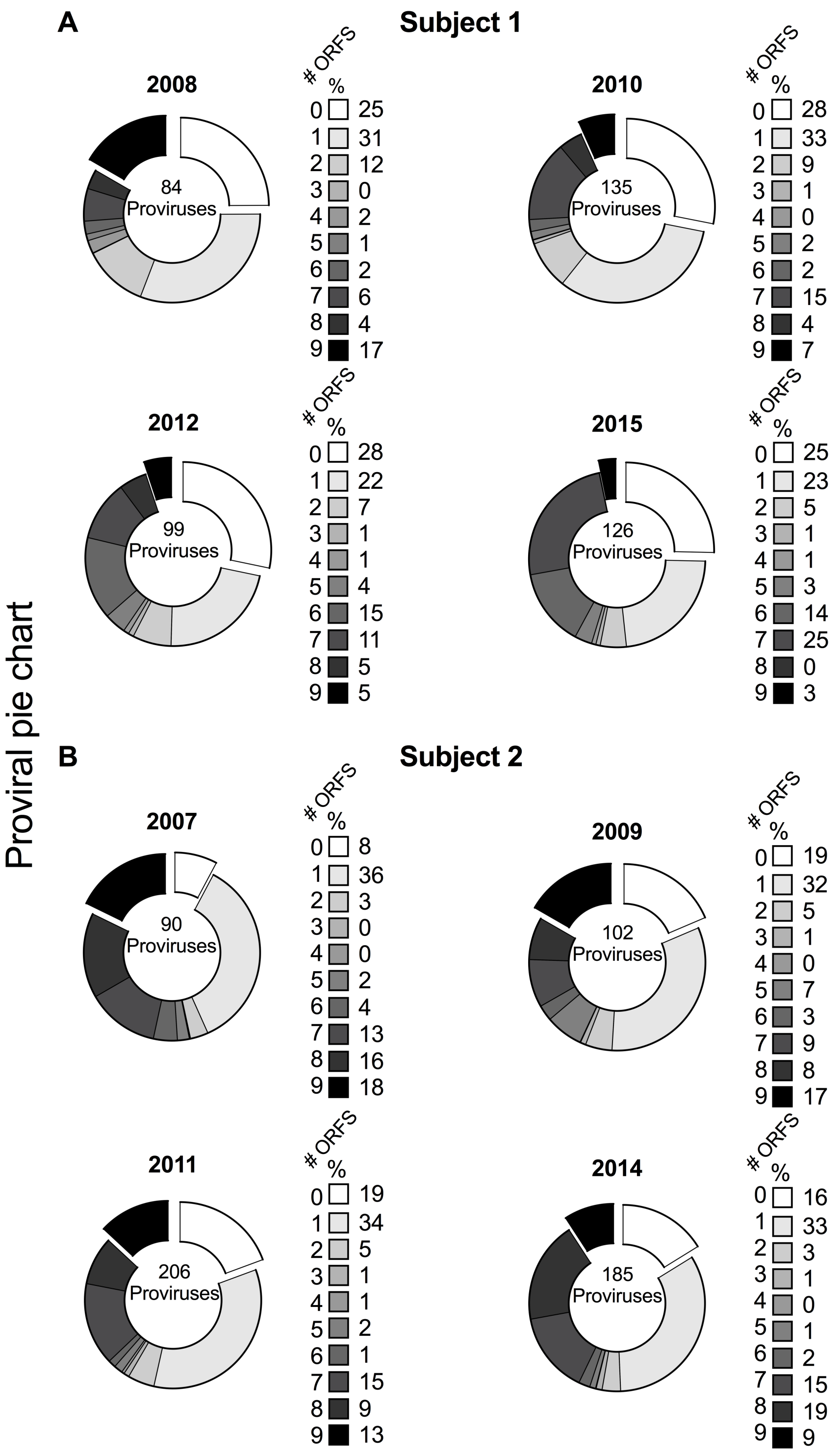

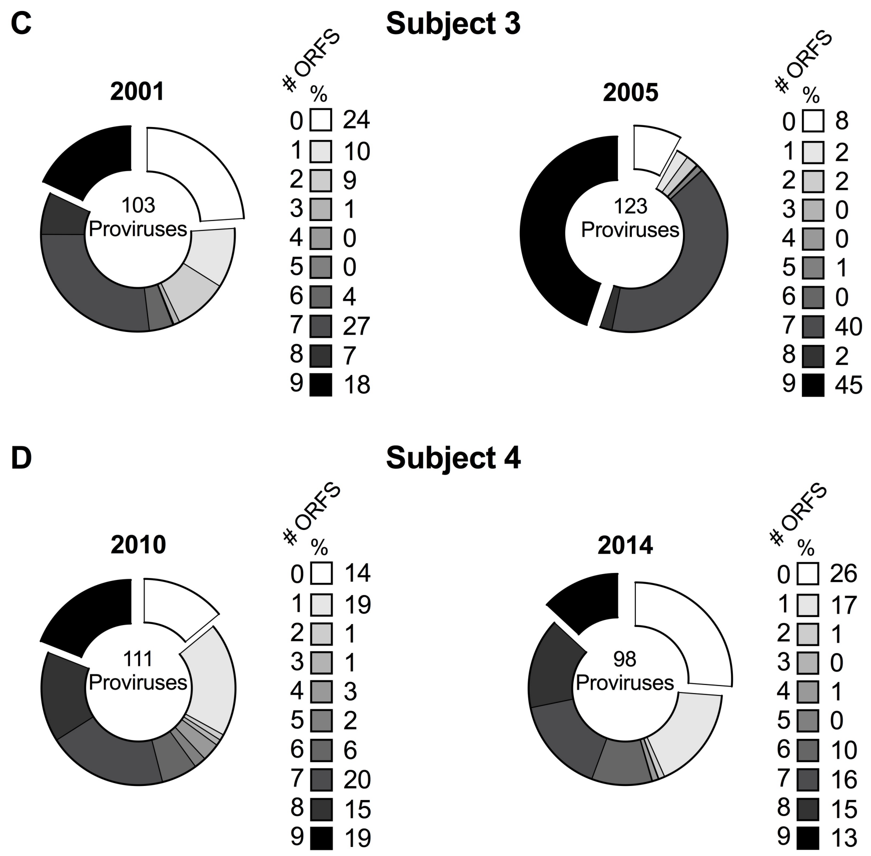

Supplementary Figure 1. Number of intact Open Reading Frames (ORFs) over time.
The total number of proviruses sequenced at each time point is reported in the center of each pie chart. For each time point, we also reported the number of proviruses containing from 0 to 9 ORFs as percentage over time.

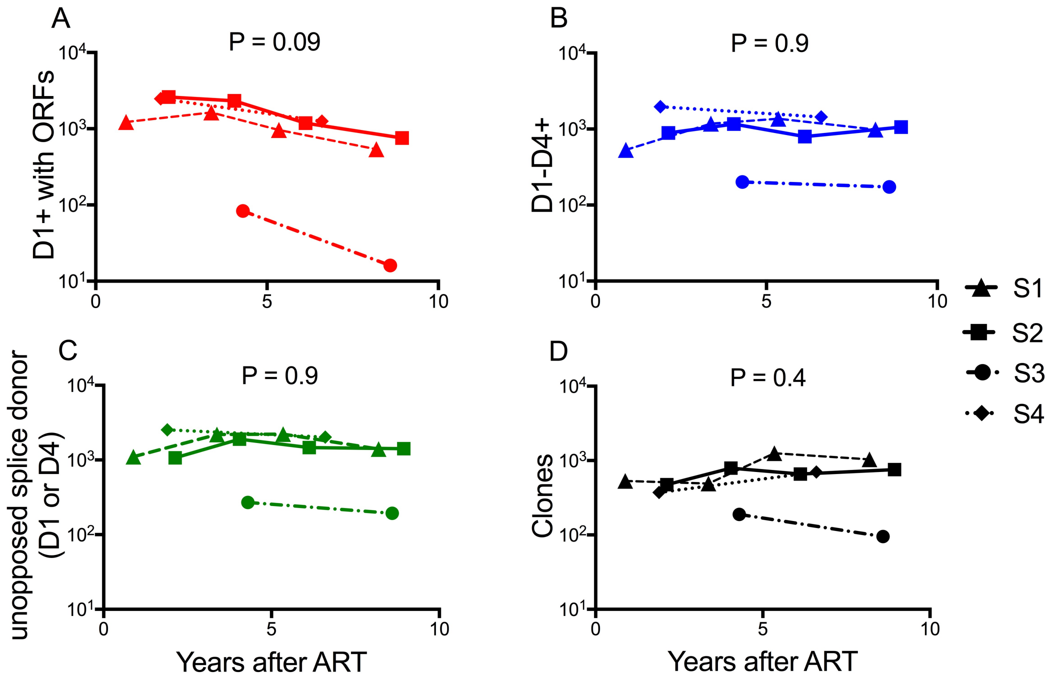

Supplementary Figure 2. Absolute changes in the major splice sites donors over time.

A) Absolute number of intact D1 splice site sequences in defective proviruses with at least one functional ORF at each time point (red). B) Absolute number of intact D4 splice sequences in defective proviruses lacking 5' D1 at each time point (blue). C) Absolute number of defective proviruses with unopposed strong donor splice site, i.e. D1+ without ORFs or D4+ without D1 (D1-D4+) at each time point (green). D) Absolute number of clones over time in defective proviruses (black). Absolute numbers are presented as log copies per million CD4 T-cells. Estimation of a common slope for the four subjects was performed using a linear random effects regression model, and assuming that each subject had a different intercept at the initiation of ART. To test for the statistical significance of this effect, a statistical analysis based on a type III Anova was performed. The number of defective proviruses containing D1 and functional ORFs showed a trend to a decline in all subjects (p=0.09 by Anova, panel A), consistent with the clearance of proviruses with the potential to express Gag/Pol. The absolute level of defective proviruses with strong donor splice site and poor potential for protein expression as well as clones did not change over time (panel B-D).

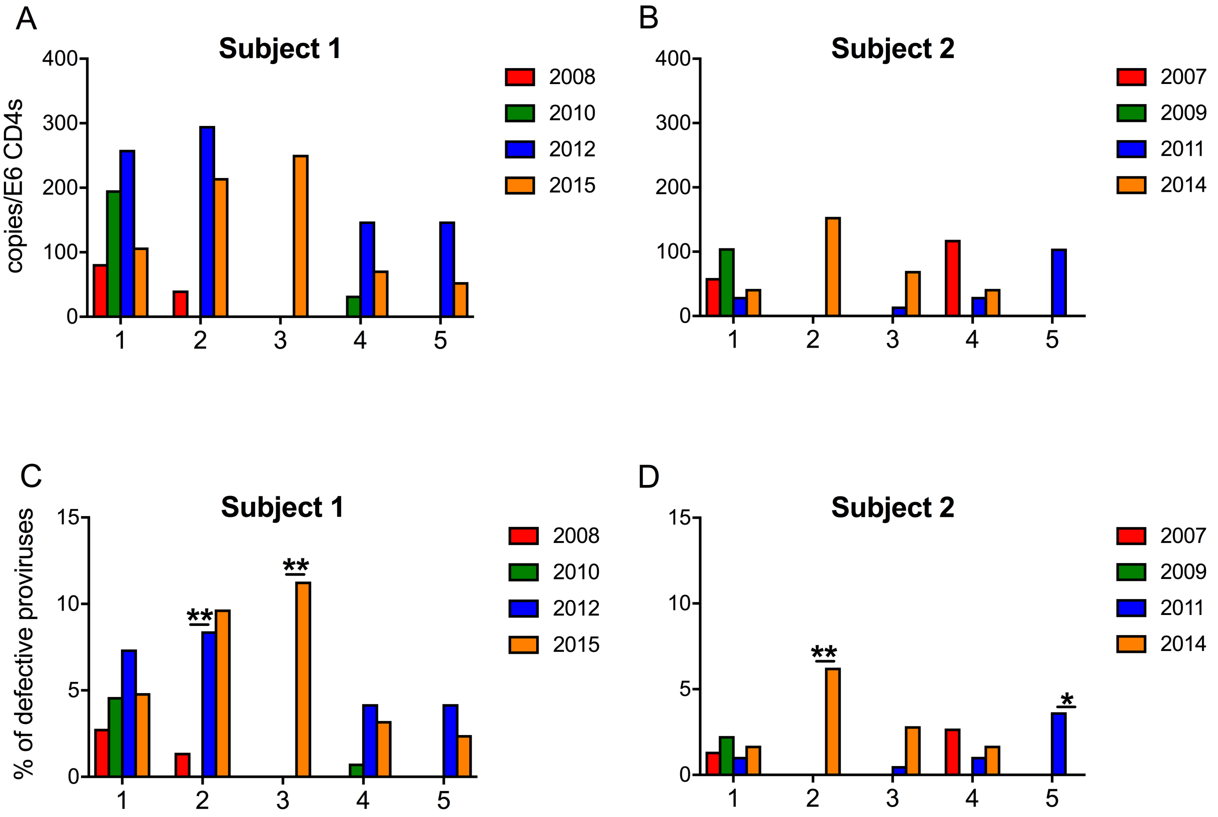

Supplementary Figure 3. Clonal expansion likely continues after ART but to a lesser extent than before ART. For Subject 1 and Subject 2, the five most prevalent clones among defective proviruses were analyzed across the four time points of the study. Clones are presented as number of copies per million CD4 T cells in panel A (Subject 1) and B (Subject 2), and as percentage of defective proviruses in panel C (Subject 1) and D (Subject 2). *$p<0.05$ **$p<0.01$. A series of binomial tests for equality of proportions were performed to detect expansion or contraction of clones over time in each clonal subspecies. In Subject 1 significant expansion of clone 2 occurred from 2010 to 2012 ($p<0.01$ by Pearson's Chi Squared Test) and clone 3 from 2012 to 2015 ($p<0.01$ by Pearson's Chi Squared Test) (panel C). In Subject 1, clone 3 appeared for the first time in 2015 and made up over 10% of defective proviruses sampled at that time point. In Subject 2 significant expansion of clone 2 occurred from 2011 to 2014 ($p<0.01$ by Pearson's Chi Squared Test) and contraction in clone 5 from 2011 to 2014 ($p=0.03$ by Pearson's Chi Squared Test) (panel D).

Supplementary Tables

Subject	Age*	Sex	Year of infection	CD4 T-cell nadir (cells/μl, year)	Viral load zenith (copies/ml, year)	Additional data
**1**	47	M	1984	295 (1998)	114,422 (1998)	First ART in 1996, but several drug holidays until June 2007
**2**	36	M	1993	0 (1999)	225,000 (2001)	First ART in 1999, but several drug holidays until July 2005
**3**	51	M	1996	14 (1997)	50,000 (1997)	---
**4**	--	F	1994	194 (2007)	74,284 (2007)	AZT monotherapy during pregnancy in 1996

ART: antiretroviral therapy; AZT: zidovudine *Age at the time of first apheresis collection

Supplementary Table 1. Clinical characteristics of the subjects enrolled in the study

						HIV DNA	Cell-associated HIV RNA			
**Subject**	**Year**	**Time on ART (yrs)**	**ART**	**Viral load (copies/ml)**	**CD4 T-cells (/μl)**	**Total (LTR)**	**Unspliced RNA+ cells (US+)**	**Multispliced RNA+ cells (MS+)**	**% US+/integrated HIV DNA**	**% MS+ in US+**
**1**	2008	0.9	ATV/r TDF/ FTC	<50	617	661	142	1.3	11.6	0.9
	2015	8.2	ATV/r TDF/ FTC	<20	718	676	96	1.4	11.4	1.5
**2**	2007	2.1	ATV/r 3TC D4T	51	629	858	189	15	29	7.9
	2015	9.9	ATV/r 3TC RAL	35	768	709	81	5	9.5	6.2
**3**	2001	4.3	AZT 3TC ABC	<50	359	48	---	---	---	---

	2005	8.6	AZT 3TC ABC	<50	337	32	---	---	---	---
**4**	2010	1.9	TDF/ FTC EFV	<50	287	641	---	---	---	---
	2014	6.6	TDF/ FTC EFV	<50	645	762	---	---	---	---

ABC: abacavir; ART: antiretroviral therapy; ATV: atazanavir; AZT: zidovudine; D4T: stavudine; EFV: efavirenz; r: ritonavir; RAL: raltegravir; TDF/FTC: tenofovir/emtricitabine; 3TC: lamivudine

Supplementary Table 2. Viro-immunological parameters, including HIV DNA and cell-associated RNA levels at two time points after ART initiation in the subjects enrolled in the study. The levels of total HIV DNA are presented as copies per million PBMCs. Cell-associated HIV RNA levels are presented as copies of unspliced (us) or multispliced (ms) RNA per million PBMCs or as a percentage of the integrated HIV DNA.

		HIV DNA/million PBMCs			HIV DNA/μl blood			HIV DNA/million CD4s			Intact/ million CD4s
**Subject**	**Year**	**Integrated (Alu-gag)**	**Total (LTR)**	**Total (gag)**	**Integrated (Alu-gag)**	**Total (LTR)**	**Total (gag)**	**Integrated (Alu-gag)**	**Total (LTR)**	**Total (gag)**	
**1**	2008	1228	661	483	3.94	2.12	1.6	6382	3437	2513	491
	2015	839	676	307	2.01	1.62	0.7	2805	2260	1026	36
**2**	2007	652	858	359	2.55	3.35	1.4	4049	5328	2227	947
	2015	855	709	330	2.39	2	0.9	3117	2585	1203	184
**3**	2001	---	48	---	---	0.15	---	---	423	---	70
	2005	---	32	---	---	0.08	---	---	225	---	97
**4**	2010	---	641	---	---	1.79	---	---	6250	---	1070
	2014	---	762	---	---	2.33	---	---	3612	---	332

Supplementary Table 3. Levels of HIV DNA and intact proviruses at two time points in the subjects enrolled in the study. Here, we provide HIV DNA levels (integrated, total measured by LTR primers and total measured by gag primers) as copies per million PBMCs, copies per million CD4 T cells, and copies per μl blood and number of intact proviruses as copies per million CD4 T cells.

		A	B	C	D	E	F
**Subject**	**Apheresis time point**	**D1 in clones with no ORFs (N)**	**D1-D4+ in clones (N)**	**A+B (N)**	**Total clones (N)**	**C/D (%)**	**Defective proviruses with D1 and no ORFs OR D1-D4+ (%)**
**1**	2008	7	3	10	13	77	38
	2010	8	2	10	15	67	52
	2012	7	22	29	34	85	63
	2015	7	41	48	58	83	63
**2**	2007	0	3	3	8	38	24
	2009	2	4	6	15	40	41
	2011	3	32	35	44	80	51
	2014	3	30	33	54	61	58

D1: Donor splice site 1; D4: Donor splice site 4; ORF: Open Reading Frame

Supplementary Table 4. Percentage of proviruses with D1 and no ORFs or D1-D4+ among the proven clones in comparison to all defective proviruses. Proven proviral clones are more likely to have strong donor splice sites that lack potential to express HIV proteins in comparison to other defective proviruses (column E *vs* column F). A significantly higher percentage of unopposed strong donors splice sequences was found among proven clones in comparison to defective proviruses (p=0.02 by Wilcoxon signed rank test), consistent with the hypothesis that splice sites may contribute to clonal expansion.

***D1**	GGTRAGT
D1a	RGTAAGA
D2	GGTGAAGGGG
D3	GGTAGGA
***D4**	AGTAAGT
A1a	TCTTAAAATTAGC (1 mismatch allowed)
A2	ATTGTTTTTCAGA (1 mismatch allowed)
A3	ATTCATTTCAGA (1 mismatch allowed)
***A4c**	TWTCATTGCCAAGT
***A4a**	TKTGYTTCWYRAMAAAAGS
***A4b**	SCTTAGG
***A5**	GCATCTCCTATGGCAGG
***A7**	YTRTCRTTBCAGA (1 mismatch allowed)
***SL1**	ACTCGGCTTGCTGARGYGCRCWCRGCAAGAGGCGAG (4 mismatches allowed)
***SL2**	CGGCGRCTGGTGAGTACGCC (2 mismatches allowed)
***SL3**	GACTAGCGGAGGCTAG (1 mismatch allowed)
***SL4**	GGTGCGAGAGCGTC (1 mismatch allowed)
***RRE**	Nucleotides 7709-8063 of HXB2 sequence (www.hiv.lanl.gov)

Supplementary Table 5. List of the sequences of splice and packaging sites used to annotate the sequenced proviruses.
All elements that were required to define a provirus as intact are indicated in bold with asterisks. The Trans-activation response element was not included as we did not capture its entire sequence with our cloning strategy. We accepted both the canonical D1 sequence (GGTRAGT) as well as a GT dinucleotide cryptic donor site located four nucleotides downstream from D1 [1] (only found in four proviruses).

Supplementary Methods 1

Criteria for excluding sequences:
To avoid ambiguous nucleotides due to low coverage at each end, we analyzed the region of each sequence from 20 nucleotides downstream of the 5' end primer to 20 nucleotides upstream of the 3' end primer.
On rare occasions, proviruses were excluded from analysis due to technical limitations. Specifically, proviruses were excluded based on the following:

1) Poor read coverage leading to assembly failure of consensus sequence
2) Reads were determined to originate from more than one provirus determined by the following criteria:
    - Dinucleotide calls (>5%) within the aligned reads, suggesting more than one provirus was present during PCR amplification. Exceptions to this rule included insertions of additional adenosine nucleotides at the beginning/end of chains with at least 5 consecutive adenosine nucleotides as well as other dinucleotide calls appearing with frequency consistent with PCR error during early rounds of amplification;
    - Regions with sharp drops in coverage suggesting the presence of a provirus with a deletion and at least one or more without the same deletion.

Supplementary Methods 2

Motifs and ORFs Identification:
Sequence reads from each provirus were *de novo* assembled to generate a consensus sequence of each proviral genome. All possible ORFs were annotated within the assembled genomes by searching for the canonical start codon sequence ATG and extending the ORF until a stop codon was reached. To be labeled as an intact HIV ORF, we required that the AUG or TTTTTT (for pol) and the stop codon to be present within 20 nucleotides of the ORF in HXB2 without premature stop codons. To identify Tat and Rev, exons 1 and 2 of Tat and Rev were annotated to the provirus genome based on 65% homology with the HXB2 Tat and Rev 1 and 2. These Tat 1/2 and Rev 1/2 homologous sequences of the provirus were then extracted, concatenated, and translated. The Tat and Rev sequences were considered intact if the sequences had no early stop codons and retained the proper stop codon. We accepted known early stops variants of Tat [2].
To identify splice and packaging sites, motifs were annotated using Geneious. They were identified as specified in Supplementary Table 5.

Supplementary References

1. Purcell, D. F. & Martin, M. A. Alternative splicing of human immunodeficiency virus type 1 mRNA modulates viral protein expression, replication, and infectivity. *J. Virol.* **67,** 6365–78 (1993).
2. Clark, E., Nava, B. & Caputi, M. Tat is a multifunctional viral protein that modulates cellular gene expression and functions. *Oncotarget* **8,** 27569–27581 (2017).
