# Supplementary material for: Longitudinal HIV sequencing reveals reservoir expression leading to decay which is obscured by clonal expansion
Source: Nat Commun. 2019 Feb 13;10:728. doi: 10.1038/s41467-019-08431-7 (PMC6374386; doi:10.1038/s41467-019-08431-7)
Supplement: Supplementary file 2 — Peer Review File [file 41467_2019_8431_MOESM2_ESM.docx]

Parts of this peer review file have been redacted as indicated to maintain the confidentiality of unpublished data.

Reviewers' comments:

Reviewer #1 (Remarks to the Author):

In the manuscript entitled “Proviral sequencing suggests the majority of the HIV reservoir is expressed over time but significant decay is obscured by clonal expansion” from Pinzone et al., used high throughput sequencing to study the proviruses of two subjects on ART at four time points over nine years. The Authors found that although intact and defective proviruses able to express viral proteins are negatively selected, those containing strong splice donor sequences are enriched over time as the result of clonal growth. Overall this work describes how the immune system and clonal proliferation dynamically influence composition of the HIV reservoir.

This is a very interesting paper that shows for the first time how the proviral forms capable of expressing HIV proteins and those with intact splice donor sites a deferentially selected during time. This is an important finding that supports the role of clonal expansion as stabilizing factor of the HIV reservoir. Give these considerations I consider this paper very positively.

Said that, however, there is one major issue that should be solved. All the conclusions of the manuscript are based on the analysis of only two patients and in many comparisons the significance is not reached decreasing the strength of their claims dramatically. Although the trend for counterselecton for protein expression proviruses in both patients is highly suggestive and logically explained by the Authors, the inclusion of at least an additional patient would be more convincing. The sequencing strategy and bioinformatics procedures are already in place and it not should take too long to add additional patients. Otherwise the Authors should provide a statistical analysis that formally supports that the number of patients and/or time points analyzed is enough to support their conclusions.

Reviewer #2 (Remarks to the Author):

The work presented by Pinzone et al is based on the hypothesis that, within the HIV reservoir, selective pressure on defective HIV DNA may be different than selective pressure on intact proviruses.

They monitored the frequency of individual proviral sequences over time, in two selected patients on suppressive ART. They employ limiting dilution PCR, followed by DNA sequencing to obtain full- length sequences on integrated HIV proviruses.

Four blood samples per patient, taken over a 8-9 period of time, have been studied.

The major criticism concerns the study of blood compartment. The evolution of viral variants analyzed only in the blood does not make it possible to describe the whole viral turnover over 8 years, whereas HIV infection is a generalized infection. Indeed, the majority of viral infection sites are in lymphoid tissues, gut, and lymph nodes, which are potential sites for residual viral replication, depending on the diffusion of antiretroviral drugs, which may be different depending on the molecules. Tissue analysis would be necessary to explore the assumptions made in this work. The analysis of blood cells, which only represents circulating cells, coming from the anatomic reservoirs via the blood and lymphatic circulations, cannot reflect the whole evolution of the viral diversity of the set of viral variants and their evolution, over 8 years. So, the conclusions presented are therefore too strong and these important limits of this work should be mentioned.

Moreover, the number of two subjects is too limited to affirm such final conclusions. Some examples:

Line 154-155: Based on the analysis: the authors indicate “A large fraction of proviruses would be capable of expressing HIV-gag”. This idea is transformed in line 61-62 where results are resumed in: “Intact proviruses are capable of HIV protein expression”!

Moreover, there is no supplementary techniques presented to explore biological properties of these intact proviruses, to confirm this assumption, based only on viral sequences.

Idem for: “We provide evidence for clonal expansion slows decay”. The clonal expansion is based on 3 clones among 6 variants. It is difficult to understand why the number of variants is so low in the phylogenetic tree, while the number of intact proviruses is higher than 6, given the number indicated line 116? So, it is also difficult to speculate on this clonal expansion only observed in only one patient out of two.

Line 262-263: “Our study is the first to show that immune pressure can play a role”. Again, the results obtained by sequencing SUGGEST an eventual role of immune pressure, but do not “SHOW” as indicated. Immune explorations are needed to confirm this assumption. It must be noted that antiretroviral therapy also plays a role by a specific ARV pressure, that needs to mentioned.

The majority of the results are descriptive and often over-interpreted, with speculations. The conclusions are too strong and need to be attenuated.

Specific comments:

Line 164: The clinical history of subject 2, with CD4 down to 0, signs a diagnosis of AIDS and indicates a late stage of HIV infection. What is the clinical history of patient 1?

The authors indicated that the two subjects have a similar history. This need to be documented for each subject, within a table, including the CD4+ nadir, the VL zenith, the date of treatment initiation,

the dates of the four samples the ARV treatment, the treatment duration including the previous ones and the time with viral control before the first sample. Lastly it could be interested to also document the values of total and integrated HIV DNA at least for the first and the last samples of each patient.

Reviewer #3 (Remarks to the Author):

This manuscript describes the longitudinal evolution of HIV proviral sequences determined by near full-length viral sequencing in two study subjects treated with ART for around 10 years. The authors conclude that viral sequences that can be effectively transcribed/translated are under negative selection pressure, while defective sequences that cannot be transcribed are positively selected for. While this is an attractive hypothesis, I have the following substantial concerns:

1. Figure 1 shows a longitudinal decrease of intact HIV sequences in the two study subjected. The authors suggest that intact viral DNA decline is faster than originally described (around 2 years, as opposed to around 44 months reported previously (i. e. Siliciano et al, Nat Med 2003, Crooks et al, JID 2015). Compared to previously published data (which included >50 study subjects), the authors' work with n=2 study subjects must be considered anecdotal. The small sample size does, from perspective, not allow to make general conclusions about the longitudinal dynamics of intact proviral sequences.
2. Regarding the defective HIV sequences, the authors distinguish proviral species that they consider as likely to be effectively transcribed/translated, and those that cannot be transcribed. This discrimination is based on theoretical considerations and not on empiric data. To make the point that these two different types of proviral species undergo different selection pressure, I believe it is critical to experimentally show differences in transcriptional activity.
3. Issue #2 is complicated by the fact that from my perspective, the longitudinal dynamics in patient 1 and 2 in Figure 3 are quite different. While the proposed hypothesis is supported by patient 1, patient 2 behaves very different. Overall, I believe it is difficult to propose such a complex hypothesis based on such small sample sizes.
4. The authors raise the important point that clonal proliferation of cells harboring proviral sequences may be dependent on transcriptional activities of oncogenes in proximity to the viral integration site (Cesana et al, Nat Com 2017). An analysis of chromosomal integration sites of clonally-expanded sequences would clearly be highly informative.

# We thank the editor and reviewers for their insightful comments and suggestions. Please find below our reply to the reviewers’queries.

Reviewers' comments:

Reviewer #1 (Remarks to the Author):

1. *All the conclusions of the manuscript are based on the analysis of only two patients and in many comparisons the significance is not reached decreasing the strength of their claims dramatically.*

Since our first submission, we have sequenced additional intact proviruses for Subject 1 and 2 and additional defective proviruses for Subject 2. Fig. 2 and 4 show the effect of including these additional data. Notably, the statistical certainty for the decay rate estimates has increased considerably. This addresses the concerns of Reviewers 2 and 3 that the data for Subject 2, in particular, was weak. Review of the paper shows that nearly all figures either have significant p values (Fig. 4A-C) or the lack of significance is predictable as explained in the paper. Notably Fig. 4D did not reach statistical significance for clonal expansion in Subject 1, but this is not a novel conclusion but rather a figure to show our data is consistent with the literature that clones increase relatively over time (a trend for Subject 1 and significant for Subject 2).

2. *Although the trend for counterselection for protein expression proviruses in both patients is highly suggestive and logically explained by the Authors, the inclusion of at least an additional patient would be more convincing…Otherwise the Authors should provide a statistical analysis that formally supports that the number of patients and/or time points analyzed is enough to support their conclusions.*

As noted above, we included two extra time points for two additional patients (Fig. S4). Statistician Wei-Ting Hwang and math modeler LaMont Cannon provided this response:

We performed a power analysis to determine the appropriate sample size from which to draw definitive conclusions regarding correlations of the entire population. Based on this power analysis, to obtain a statistical power of 0.8 with a significance level of 0.05 and to distinguish a small effect size of 0.1 (Cohen 1988), we would need to analyze at least 782 subjects. Even when the effect size relaxed to the largest suggested value of 0.5 (Cohen 1988), we would still be required to analyze at least 29 patients in order to reach the desired statistical power. It is not feasible to gather longitudinal data and perform the subsequent analysis for this number of subjects. Rather the aim for this study is to demonstrate two patients with dissimilar reservoir decay rates and to provide potential explanations to explain this variation. Nonetheless, we did include two data points from two additional subjects which continue to support our major hypotheses. Sequencing is costly in both time and money and as explained above we have not yet found any patients with samples obtained over a similar time course. After reflection on the reviewers’ insightful comments, we envision the best way to evaluate if our results are generalizable

would be to study 3 cohorts with variable ability to exert immune pressure (elite controllers, acute and chronic individuals) on ART. While we are convinced this is a worthy study to pursue, it is beyond the scope of this manuscript.

Reviewer #2 (Remarks to the Author):

1. *The major criticism concerns the study of blood compartment…The analysis of blood cells, which only represents circulating cells, coming from the anatomic reservoirs via the blood and lymphatic circulations, cannot reflect the whole evolution of the viral diversity of the set of viral variants and their evolution, over 8 years. So, the conclusions presented are therefore too strong and these important limits of this work should be mentioned.*

We agree with Reviewer 2 that this potential limitation of our study should be mentioned in our discussion. Notably, recent work suggests that there is significant mixing between the blood and lymph which, taken together, represents a sizable fraction of the total body lymphocyte pool (Di Mascio et al., 2009). Furthermore, recently Kearney showed that these two phylogenetic compartments (blood and lymph nodes) are not distinguishable [(Kearney et al., 2015; Wiegand et al., 2017) and CROI, 2018]. We added the following text.

“An important limitation of our study is the small number of subjects studied as well as the fact that our analysis is comprised of only the circulating white blood cells. Thus, our data do not reflect the selection of proviruses in tissue-resident lymphocytes that do not recirculate. However, as recent studies have shown that the phylogeny of proviruses isolated from lymph nodes and blood is similar, our analysis likely reflects selection pressures exerted on the circulating pool of lymphocytes that travel between the blood, spleen and lymph nodes (Kearney et al., 2015; Wiegand et al., 2017).”

2. *Moreover, the number of two subjects is too limited to affirm such final conclusions.*

We do agree that we must temper our conclusions and we have. Please see the response from our math modeler and statistician for Reviewer 1 explaining our rationale for only studying two patients in depth but adding two time points from two additional subjects and adding substantially more time points and sequences from Subject 1 and 2. Thus, the decay curves of Subject 2 (as well as Subject 1) now also reaches statistical significance in the current manuscript.

3. *Some examples:*

*Line 154-155: Based on the analysis: the authors indicate “A large fraction of proviruses would be capable of expressing HIV-gag”. This idea is transformed in line 61-62 where results are resumed in: “Intact proviruses are capable of HIV GAG protein expression”!*

We have carefully revised the text to be consistent in our description both in the results and discussion section. The specific objectionable phrases have been removed.

For example, we changed:

*“A large fraction of proviruses would be capable of expressing HIV-gag”*

To

“Interestingly, we found that a large fraction of these proviruses had the genetic potential to express HIV Gag and occasionally HIV Pol..”

And we deleted: *“Intact proviruses are capable of HIV GAG protein expression”!*

4. *Moreover, there is no supplementary techniques presented to explore biological properties of these intact proviruses, to confirm this assumption, based only on viral sequences.*

We have done additional experiments to explore the biological properties of the proviruses making up the reservoir of the two subjects. Notably, we demonstrated the intact clone that emerged after 9 years in Subject 2 is replication-competent by quantitative viral outgrowth assay (Fig. 2D). Additionally, our sequence-based criteria for intact proviruses were slightly more stringent than those used by Ho et al., 2013. Therefore, it is reasonable to conclude that our criteria for considering a provirus intact could be at least as rigorous as those used in (Ho et al., 2013) to identify intact, likely replication-competent, proviruses.

5. *Idem for: “We provide evidence for clonal expansion slows decay”. The clonal expansion is based on 3 clones among 6 variants. It is difficult to understand why the number of variants is so low in the phylogenetic tree, while the number of intact proviruses is higher than 6, given the number indicated line 116?*

*So, it is also difficult to speculate on this clonal expansion only observed in only one patient out of two.*

To increase the sample size in our phylogenetic analysis, we have sequenced more intact proviruses. For Subject 2, we have now identified 9 clones, one of which is made up of 26 proviruses, including the ones sequenced from the QVOA supernatant. For Subject 1, we have identified 5 identical sequences that constitute two clones (Fig. 2C-D).

6. *Line 262-263: “Our study is the first to show that immune pressure can play a role”. Again, the results obtained by sequencing SUGGEST an eventual role of immune pressure, but do not “SHOW” as indicated. Immune explorations are needed to confirm this assumption. It must be noted that antiretroviral therapy also plays a role by a specific ARV pressure, that needs to mentioned. The majority of the results are descriptive and often over-interpreted, with speculations. The conclusions are too strong and need to be attenuated.*

We agree that we should temper our conclusions. We have done so throughout the manuscript. We also agree with the reviewer that it would be important to explore the

contribution of CTL and provide preliminary *ex vivo* data consistent with our hypothesis that immune pressure was stronger in Subject 1 than Subject 2.

[Redacted]

After embarking on these experiments, we came to understand the complexity of probing immune pressure and believe this question is better left for a future study where we intend to study proviral sequences over time in acutely infected, chronically infected, and elite controllers placed on ART. However, this is beyond the scope of this manuscript and the current resources of my laboratory.

[Redacted]

Notably, there is a parallel between our *in vivo* and *in vitro* data. Subject 1 experienced a significant drop in the levels of HIV DNA and *in vivo* this subject experienced a two- fold drop in DNA whether normalized by CD4 count or volume, while Subject 2 did not.

We agree that ART may exert a specific pressure. We do not have pharmacokinetic data in our subjects, and we cannot rule out that some rounds of ongoing replication (either due to poor ARV penetration into some compartments or drug resistance) may have occurred. We have added the following statement to the discussion.

“We cannot rule out that some rounds of ongoing replication (either due to poor ART penetration into some compartments or drug resistance) may have occurred, especially in Subject 2.”

However, we have analyzed the intact sequences and discovered that all the intact proviruses sequenced remained sensitive to the antiviral regimen of Subject 1 and 2.

7. Specific comments:

*Line 164: The clinical history of subject 2, with CD4 down to 0, signs a diagnosis of AIDS and indicates a late stage of HIV infection. What is the clinical history of patient 1?*

*The authors indicated that the two subjects have a similar history. This need to be documented for each subject, within a table…Lastly it could be interested to also document the values of total and integrated HIV DNA at least for the first and the last samples of each patient.*

We thank the reviewer for this insightful comment. On deeper reflection, the two individuals do not have similar clinical histories. We have now included supplementary tables with the additional clinical data requested by Reviewer 2 (Table S1, S2 and S3). Subject 2 clearly progressed much faster than Subject 1 and this is central to our hypothesis.

Reviewer #3 (Remarks to the Author):

1. *The small sample size does, from my perspective, not allow general conclusions about the longitudinal dynamics of intact proviral sequences.*

We did not intend to say our method represents an estimate of the average decay of the reservoir at a population level. We intended to say our method provides an estimate of reservoir decay for two individuals. Moreover, even the cited studies from the Siliciano Lab (Finzi et al., 1999; Siliciano et al., 2003) showed that every patient had a different decay. Some subjects even had reservoir expansion, while other subjects had an even faster decay of intact proviruses than the ones we studied. We estimated the half-life to be approximately 1.8 years for Subject 1 and 3.4 years for Subject 2. These values are well within the confidence interval of 17.4-133.2 months established in the study from the Siliciano Lab.

2. *Regarding the defective HIV sequences, the authors distinguish proviral species that they consider as likely to be effectively transcribed/translated, and those that cannot be transcribed. This discrimination is based on theoretical considerations and not on empiric data. To make the point that these two different types of proviral species undergo different selection pressure, I believe it is critical to experimentally show differences in transcriptional activity.*
A) [Redacted]
B) In the resubmitted manuscript, we show additional evidence of splicing between HIV proviruses and downstream exons (Fig. 5). Importantly, we show for the first time that a variety of chimeric transcripts between D4 and downstream exons are made in *in vitro* infected cells.
C) We now show that the frequency of HIV Gag RNA expression ranges from 10-30% (Table S2). We cannot distinguish if these are replication-competent or not, but given that the level of replication-competent infected cells dropped below 1% at the latest timepoint surely transcription of defectives is also occurring as supported by the work

of Mary Kearney [(Kearney et al., 2015; Wiegand et al., 2017) and CROI, 2018]. This is consistent with other papers that have experimentally shown that defective HIV can frequently be transcribed (Imamichi et al., 2016; Kearney et al., 2015; Wiegand et al., 2017) as well as translated (DeMaster et al., 2015). We do not necessarily claim that all intact HIV genes are transcribed. Instead, we hypothesize that transcription of defective proviruses would provide a reasonable mechanism for positive selection when splicing to a downstream exon might induce cell division and negative selection when translation of HIV ORFs might lead to cell death.

3. *Issue #2 is complicated by the fact that from my perspective, the longitudinal dynamics in patient 1 and 2 in Figure 3 are quite different…Overall, I believe it is difficult to propose such a complex hypothesis based on such small sample sizes.*

We sequenced additional proviruses for Subject 2, which increased the strength of our analysis. Moreover, to clarify, Subject 2 does also support the hypothesis. However, in his case D1+D4+ (black) defective proviruses are the category that is cleared more over time in comparison to D1+D4- (red) defective ones (Fig. 3).

4. *An analysis of chromosomal integration sites of clonally-expanded sequences would clearly be highly informative.*

We thank the reviewer for his insightful suggestions and agree that this is a worthy and challenging suggestion. While there are many techniques for cloning proviruses, current techniques do not provide chromosomal addresses for specific proviral sequence because too little of the HIV genome is sequenced to allow firm identification of the specific provirus. For this question, we did perform RNA sequencing and screened for chimeric transcripts between D1 and downstream oncogenes and between D4 and downstream oncogenes. We identified 20 chimeric D4 human exon transcripts and 1 chimeric D1 human exon (Fig. 5). The predominance of D4 over D1 also bolsters our hypothesis.

Reviewers' comments:

Reviewer #1 (Remarks to the Author):

In the revised version of this manuscript the Authors have improved the statistical analyses by adding additional data. The hypothesis is sound and the data produced appear to suggest that the Authors are on the right path to convincingly sustain that (as quoted) "HIV reservoir is expressed over time but significant decay is obscured by clonal expansion". However, despite the improvements, the Authors still base their claims on the study to just two subjects, which is really risky.

I understand that this type of analyses hundreds of subjects would be required to achieve true statistical significance (as admitted by the Authors). It would be maybe unfair to as for such an effort, however, the inclusion of at least one or two additional subjects would be helpful to gain at least a little bit more confidence over Author's claims.

It is unclear why it is so difficult to find samples from additional patients with similar characteristics of those used in this study. Is because the patients must be strictly matched in terms of therapeutic regimen? If yes, can the authors justify the stringency adopted for the selection of patients?

If I am not wrong, Authors argue that the costs for including additional patients would be prohibitive. However, considering the costs of NGS sequencing in these now days, I disagree that the cost for including an additional patient would be so prohibitive. Moreover, cost issues do not help anyway on stats.

Therefore, in this Reviewer's opinion, the study of just two subjects in the study appears to be still a major issue that needs to be addressed (and that could be addressed actually) in order to justify Author's claims. The Authors should add additional patients and confirm or drop the claim on the counter selection, which would be a pity since its the main claim of the paper.

Reviewer #3 (Remarks to the Author):

The manuscript has been improved, I have no more comments at this stage. Further comments regarding issues raised by Reviewer #2:

The authors have responded to Rev 2 by including more sequencing data on subjects 1 and 2, and integrating data from two more study subjects in supplemental Figure S4. The number of intact and defective sequences entered in the analysis in Figure S4 (for each timepoint) should be specified, and it would be helpful if the data for subject 3 and 4 are summarized in a genome structure diagram similar to what is shown Figure 3. Use of blood-derived cells (as opposed to lymphoid tissue-derived cells) is now identified as a limitation of the study, and in my opinion, it is ok to defer analysis of tissue reservoirs by full-genome sequencing to future studies.

## Reviewer #1 (Remarks to the Author):

*In the revised version of this manuscript the Authors have improved the statistical analyses by adding additional data. The hypothesis is sound and the data produced appear to suggest that the Authors are on the right path to convincingly sustain that (as quoted) "HIV reservoir is expressed over time but significant decay is obscured by clonal expansion".*

*However, despite the improvements, the Authors still base their claims on the study to just two subjects, which is really risky.*

. *.. .however, the inclusion of at least one or two additional subjects would be helpful to gain at least a little bit more confidence over Author's claims.*

We thank Reviewer 1 for pushing us on this issue. We now analyze 4 patients in figures 1, 4, and 5. Figure 4 and 5 provide the data central to our hypothesis that there are two opposing forces. In retrospect, it was an error to not show the two additional patients we analyzed directly into Figure 4 in our resubmission. Regretfully, we simply added these two patients as a buried supplement (Supplemental Figure 4). In the current resubmission, we include these two additional patients in the main Figure 4 and 5. Until recently, I did not realize that I could use "mixed linear regression effects" to combine all the data. The comments from Reviewer 1 inspired me to talk to a statistician - a benefit of the review process!

## Reviewer #3 (Remarks to the Author):

*The manuscript has been improved, I have no more comments at this stage. Further comments regarding issues raised by Reviewer #2:*

*The authors have responded to Rev 2 by including more sequencing data on subjects 1 and 2, and integrating data from two more study subjects in supplemental Figure S4.*

*The number of intact and defective sequences entered in the analysis in Figure S4 (for each timepoint) should be specified, and it would be helpful if the data for subject 3 and 4 are summarized in a genome structure diagram similar to what is shown Figure 3.*

In Supplemental Figure S1, we specify the number of proviruses sequenced for Figure 4 and 5 for all subjects.

We thank Reviewer 3 for his suggestion to also provide a deletion map of the two additional subjects as this indeed bolsters our hypothesis and strengthens our claims. The old Figure 3 and 4 now correspond to Figure 4 and 5 in our resubmitted manuscript.

Sincerely, Una
